# TREM2 suppresses the proinflammatory response to facilitate PRRSV infection via PI3K/NF-κB signaling

**DOI:** 10.1371/journal.ppat.1008543

**Published:** 2020-05-13

**Authors:** Zhenbang Zhu, Xiaoxiao Zhang, Wenjuan Dong, Xiaoying Wang, Sheng He, Hui Zhang, Xun Wang, Ruiping Wei, Yaosheng Chen, Xiaohong Liu, Chunhe Guo

**Affiliations:** State Key Laboratory of Biocontrol, School of Life Sciences, Sun Yat-sen University, Guangzhou Higher Education Mega Center, Guangzhou, Guangdong, PR China; Research Center for Molecular Medicine, AUSTRIA

## Abstract

Triggering receptor expressed on myeloid cells 2 (TREM2) serves as an anti-inflammatory receptor, negatively regulating the innate immune response. TREM2 is mainly expressed on dendritic cells and macrophages, the target cells of porcine reproductive and respiratory syndrome virus (PRRSV). Thus, we investigated the potential role of TREM2 in PRRSV infection in porcine alveolar macrophages (PAMs). We found that there was an increased expression of TREM2 upon PRRSV infection *in vitro*. TREM2 silencing restrained the replication of PRRSV, whereas TREM2 overexpression facilitated viral replication. The cytoplasmic tail domain of TREM2 interacted with PRRSV Nsp2 to promote infection. TREM2 downregulation led to early activation of PI3K/NF-κB signaling, thus reinforcing the expression of proinflammatory cytokines and type I interferons. Due to the enhanced cytokine expression, a disintegrin and metalloproteinase 17 was activated to promote the cleavage of membrane CD163, which resulted in suppression of infection. Furthermore, exogenous soluble TREM2 (sTREM2)-mediated inhibition of PRRSV attachment might be attributed to its competitive binding to viral envelope proteins. In pigs, following PRRSV challenge *in vivo*, the expression of TREM2 in lungs and lymph nodes as well as the production of sTREM2 were significantly increased. These novel findings indicate that TREM2 plays a role in regulating PRRSV replication via the inflammatory response. Therefore, our work describes a novel antiviral mechanism against PRRSV infection and suggests that targeting TREM2 could be a new approach in the control of the PRRSV infection.

## Author summary

Porcine reproductive and respiratory syndrome virus (PRRSV) is one of the most important pathogens in the global swine industry and is difficult to control. CD163 is a receptor that is required for PRRSV infection, and its expression is regulated by various inflammatory mediators. Triggering receptor expressed on myeloid cells 2 (TREM2) is a novel immunoregulatory receptor, which is involved in innate immunity and anti-inflammatory responses. Here we sought to elucidate whether TREM2 regulates CD163 expression to promote PRRSV infection via the inflammatory response. We found increased TREM2 levels upon PRRSV infection *in vitro* and *in vivo* in the lungs and lymph nodes of infected pigs. We demonstrated that the cytoplasmic tail of TREM2 interacts with PRRSV Nsp2 to promote infection. Additionally, TREM2 silencing triggers an early proinflammatory response via PI3K/NF-κB pathway, thus downregulating the expression of CD163, which leads to virus suppression. Our work provides a novel antiviral mechanism against PRRSV infection and suggests that targeting TREM2 could be a new approach for control of the disease.

## Introduction

Porcine reproductive and respiratory syndrome virus (PRRSV) is one of the most prominent swine diseases in the world [1, 2]. It was first reported in the late 1980s in the United States of America, followed by outbreaks in many countries and regions immediately thereafter [3]. PRRSV is a single-stranded, positive-sense RNA virus with a genome size of 15.4 kb, containing at least 10 open reading frames (ORFs) [4, 5]. PRRSV has a highly restricted cell tropism both *in vitro* and *in vivo*, with porcine alveolar macrophages (PAMs) as the primary target [6, 7]. The receptors on the cell surface play an important role in PRRSV infection [8]. CD163 has been shown to serve as a receptor participating in the uncoating of the virion [9–11]. Blocking or downregulating the expression of CD163 can restrict PRRSV infection [12–14]. It is reported that various stimuli and factors regulate CD163 expression *in vitro*. Glucocorticoids, interleukin (IL)-6, and IL-10 positively stimulate CD163 expression, whereas IL-1α, IL-1β, IL-8, IL-4, lipopolysaccharide (LPS), TNF-α, interferon γ, CXC-chemokine ligand 4 (CXCL4), and even Toll-like receptor 2/4/5 (TLR2/4/5) downregulate the expression of CD163 [15, 16]. A disintegrin and metalloproteinase 17 (ADAM17) was identified as an enzyme responsible for the cleavage of CD163 in macrophages [17, 18]. Inflammatory *in vivo* and *in vitro* conditions may lead to the activation of ADAM17 and subsequent reduction of CD163 [19, 20].

Triggering receptor expressed on myeloid cells 2 (TREM2) is a novel cell surface receptor, which was first identified in 2000 [21]. It is a member of the TREM transmembrane glycoprotein family and is widely distributed on the surface of macrophages, microglia, dendritic cells, and osteoclast precursors [22, 23]. The TREM2 receptor is composed of an Ig-like V-type extracellular domain, a transmembrane region, and a short cytoplasmic tail. TREM2 associates with DNAX activation protein of 12 kDa (DAP12), a signaling adaptor molecule with an immunoreceptor tyrosine-based activation motif (ITAM) in its cytoplasmic domain, for the delivery of intracellular signals and regulation of cell function [24, 25]. TREM2 functions as an immunomodulatory receptor, which negatively regulates inflammation [26–28]. It has the potential to inhibit the inflammatory response through the suppression of TLR or PI3K/NF-κB/JNK signaling [25, 29–33]. TREM2 is also associated with many infectious diseases, including bacterial, viral, and parasitic infections [34, 35]. It was reported that TREM2 could protect the corneas of BALB/c mice from *Pseudomonas aeruginosa* infection by weakening corneal inflammation and pyroptosis [36]. TREM2 is involved in the malaria liver stage infection through the activation of Kupffer cells [37]. Holtzman’s group reported that Sendai virus increases the levels of TREM2 and soluble TREM2 (sTREM2) in lung macrophages, which protects macrophages from apoptosis, facilitating a shift from acute infection to chronic inflammatory disease after respiratory viral infection [38]. Mice lacking TREM2 are protected from lymphocytic choriomeningitis virus-induced hepatitis and show improved virus control [39]. The human herpesvirus 6A (HHV-6A) infection induces the activation of TREM2 and migration of microglial cells [40]. TREM-2 expression is significantly increased in alveolar monocytes/macrophages of influenza virus-infected pigs [41]. Adeno-associated virus infection induces microglial TREM2 overexpression in the hippocampus of wild-type mice [42]. DAP12 knockdown also intensifies PRRSV-induced proinflammatory cytokine production and inhibits viral replication [43]. However, little is known about TREM2 in the regulation of PRRSV infection.

In the current study, we determined the role of TREM2 in PRRSV infection and elucidated the underlying mechanisms of TREM2-mediated PRRSV infection. We demonstrate that the expression of TREM2 has a positive correlation with PRRSV infection *in vivo* and *in vitro*. TREM2 and its downstream signaling negatively regulated the host proinflammatory response and enhanced the expression of CD163, which contributed to PRRSV infection. The cytoplasmic tail domain of TREM2 was responsible for promoting the replication of PRRSV via binding to PRRSV Nsp2. Moreover, exogenous sTREM2 could restrain PRRSV attachment to the cell surface through interaction with PRRSV envelope proteins.

## Results

### TREM2 facilitates PRRSV infection

**Fig 1:**
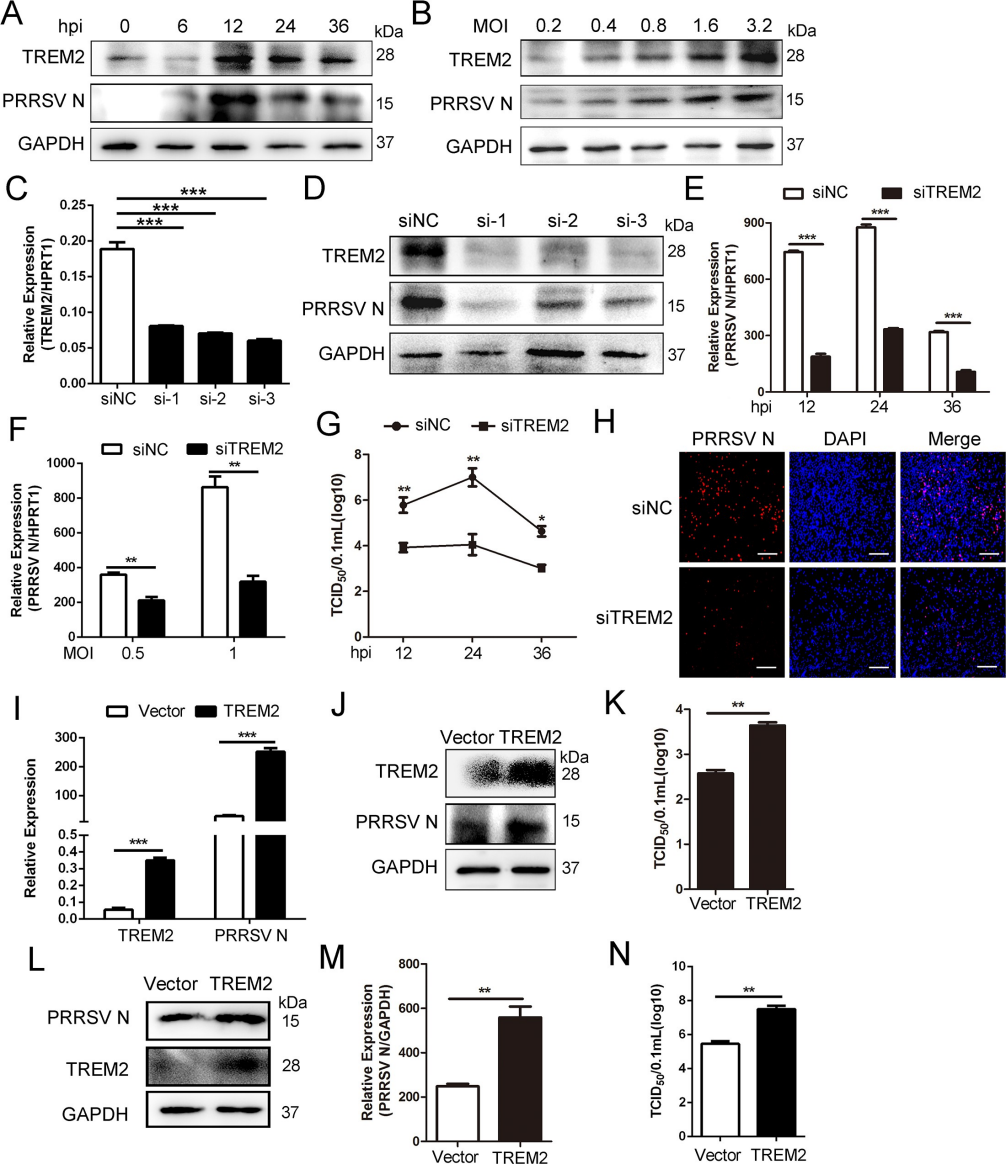
TREM2 contributes to the replication of PRRSV. (A) PAMs were infected with PRRSV (MOI = 1) for the indicated periods or (B) at different MOIs for 24 h, protein levels of TREM2, PRRSV N, and GAPDH are shown, as detected by western blot using the indicated antibodies. GAPDH is shown as an internal control. (C to H) TREM2 knockdown restrains PRRSV infection. (C) PAMs were transfected with scrambled siRNA (siNC) or three TREM2 siRNAs (siTREM2) for 24 h, gene expression of TREM2 is shown using qRT-PCR analysis. (D) PAMs were transfected with siNC or three siTREM2 for 24 h and then infected with PRRSV (MOI = 1) for 24 h, protein levels of TREM2 and PRRSV N are shown, as detected by western blot. (E and F) PAMs were treated with siNC or siTREM2-1 and additionally infected with PRRSV (MOI = 1) for the indicated time points (E) or at different MOIs (F), the expression of viral ORF7 (PRRSV N) is shown, as detected by qRT-PCR. (G) Cell supernatants from different periods post-infection were collected for TCID_50_. (H) PAMs were treated as described in E, viral load at 24 h post-infection is shown using immunofluorescence analysis (Bar, 200 μm). (I—N) TREM2 overexpression facilitates the replication of PRRSV. PAMs or Marc-145 cells were transfected with pcDNA3.1-control (vector) or pcDNA3.1-TREM2 for 24 h, and then infected with PRRSV (MOI = 1) for another 24 h. PAMs were collected and the transcription levels of TREM2 and PRRSV N are shown using qRT-PCR (I); protein levels are shown using western blot analysis (J). (K) Viral production in PAMs was measured and is shown as TCID_50_. (L-N) Marc-145 cells were collected; protein levels of TREM2 and PRRSV N are shown (L), transcription levels are shown (M) and the TCID_50_ is shown from cell supernatants (N)_._ Data are representative of the results of three independent experiments (mean ± SE). Significant differences are indicated as follows: * (*P* < .05), ** (*P* < .01) and *** (*P* < .001).

PAMs are the primary targets of PRRSV [44]. TREM2 is widely expressed on macrophages and dendritic cells and serves as an immunoregulatory receptor [45]. Accordingly, we investigated the role of TREM2 in PRRSV infection. First, the kinetics of TREM2 expression in PAMs were analyzed by western blot at various hours post-infection (hpi) or multiplicities of infection (MOIs) following PRRSV infection. There were increased levels of TREM2 mRNA and protein in PAMs, concomitant with PRRSV replication (Fig 1A, 1B and S1A and S1B Fig). Subsequently, three siRNAs were designed for reducing the expression of TREM2 (Fig 1C). TREM2 knockdown suppressed PRRSV replication as shown by decreased levels of viral nucleocapsid (N) protein (Fig 1D). Similarly, viral ORF7 mRNA was also markedly reduced at 12, 24, and 36 hpi following TREM2 knockdown (Fig 1E). Silencing of TREM2 also inhibited PRRSV replication at different MOIs (Fig 1F). When virus titers were measured at different hours post PRRSV infection, the results showed that TREM2 silencing induced lower virulence at 12, 24, and 36 hpi compared to controls (Fig 1G). Immunofluorescence analysis also demonstrated a significant inhibition of viral replication in PAMs with TREM2 knockdown (Fig 1H). To identify whether overexpression of TREM2 had the opposite effect on PRRSV infection, TREM2 was overexpressed in PAMs and Marc-145 cells. As expected, TREM2 overexpression facilitated the replication of PRRSV and the expression of PRRSV N protein both in PAMs (Fig 1I and 1J) and Marc-145 cells (Fig 1L and 1M). Compared with the control vector, high expression of TREM2 significantly enhanced viral yields in both PAMs (Fig 1K) and Marc-145 cells (Fig 1N). Taken together, TREM2 facilitates the replication of PRRSV.

### The cytoplasmic tail domain of TREM2 upregulates PRRSV replication through interaction with PRRSV Nsp2

**Fig 2:**
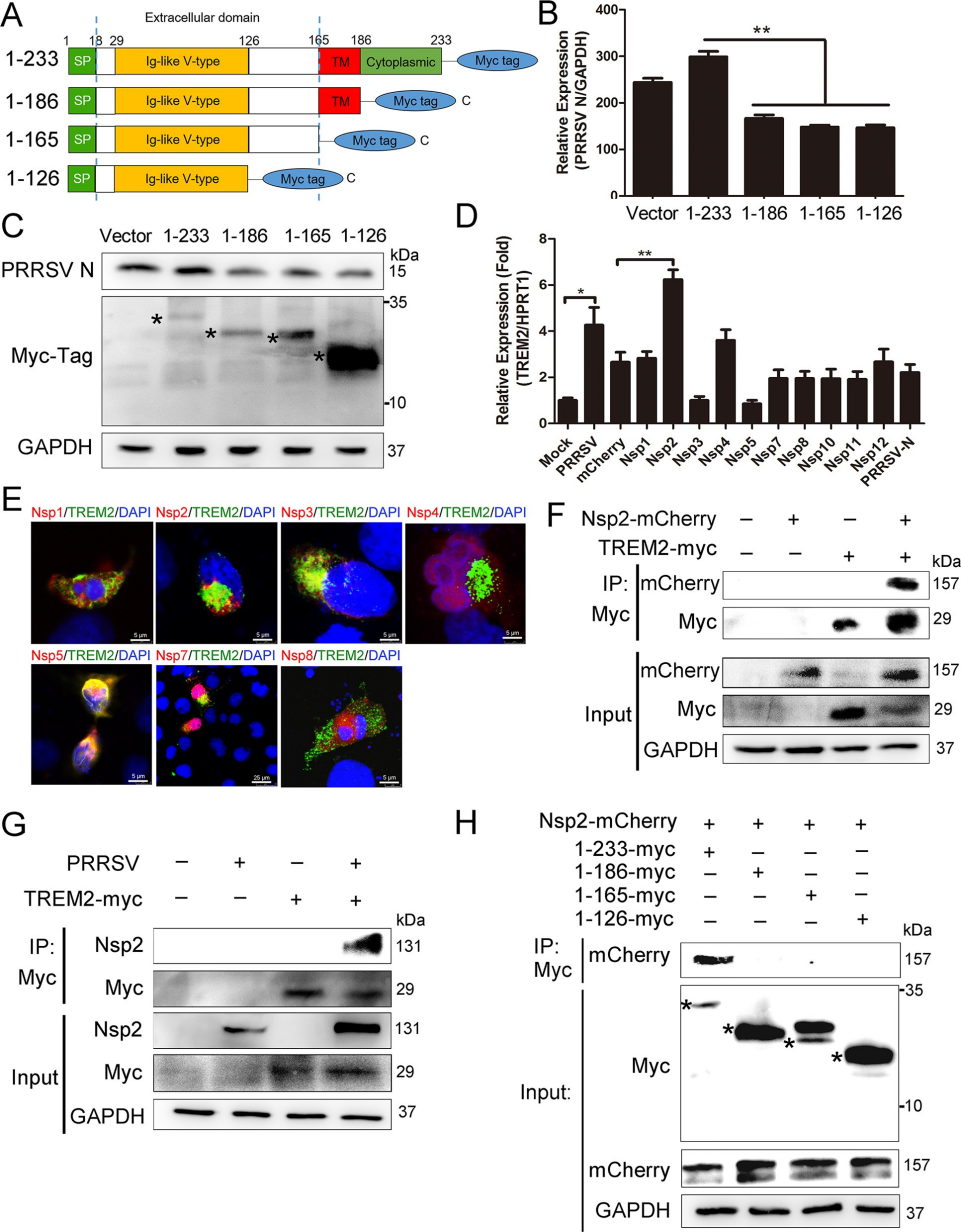
The cytoplasmic tail domain of TREM2 facilitates PRRSV replication via interaction with PRRSV Nsp2. (A) Schematic representations of full-length (aa1–aa233) (designated 1–233) or truncated (designated 1–186, 1–165 and 1–126) TREM2 constructs, all tagged with Myc at the C-terminus. SP, signal peptide; TM, transmembrane domain. (B and C) Full-length TREM2 or other TREM2 fragments were transfected into Marc-145 cells. After overexpression for 24 h, cells were infected with PRRSV (MOI = 1) for 24 h. The transcription levels (B) and protein levels (C) of PRRSV N are shown, as analyzed by qRT-PCR and western blot, respectively. Asterisks mark the expressed Myc-fusion proteins of full-length or other truncated TREM2. GAPDH is shown as an internal control. (D) mCherry control vector and ten mCherry-tagged viral NSPs were transfected into PAMs for 36 h, respectively. The transcription of TREM2 is shown, as measured by qRT-PCR. (E) Myc-tagged TREM2 and the different mCherry-tagged NSPs were co-transfected into HEK293T for 24 h. Immunofluorescence analysis of the co-localization between TREM2 (green) and NSPs (red) is shown. (Bar, 5 μm). (F) Myc-tagged TREM2 and mCherry-tagged Nsp2 were separately transfected or co-transfected into HEK293T for 36 h. Cell lysates were immunoprecipitated with Myc antibody, and the immunoblots with Myc and mCherry antibodies are shown. GAPDH is shown as an internal control. (G) Myc-tagged TREM2 was transfected into Marc-145 cells for 24 h and infected with PRRSV (MOI = 1) for 24 h. TREM2 was immunoprecipitated with Myc antibody and immunoblots with PRRSV Nsp2 and Myc antibodies are shown. GAPDH is shown as an internal control. (H) mCherry-tagged Nsp2 was co-transfected with Myc-tagged full-length or other truncated TREM2 in HEK293T. IP assays with anti-Myc antibody were performed to determine their interaction; the Nsp2-mCherry immunoblot with anti-mCherry antibody is shown. GAPDH is shown as an internal control. Asterisks mark the expressed Myc-fusion proteins of full-length or other truncated TREM2. Data are representative of the results of three independent experiments (mean ± SE). Significant differences are indicated as follows: * (*P* < .05), ** (*P* < .01) and *** (*P* < .001).

To map the domain(s) of TREM2 associated with PRRSV replication, Marc-145 cells were transfected with Myc-tagged full-length or truncated TREM2 and then infected with PRRSV (Fig 2A). We found that TREM2 fragments lacking the cytoplasmic tail region did not influence the expression of PRRSV N, suggesting that the TREM2 cytoplasmic tail domain is required for regulation of PRRSV (Fig 2B and 2C). Next, we screened the non-structural proteins (NSPs) of PRRSV responsible for the upregulation of TREM2. Ten mCherry-tagged NSPs were transfected into PAMs, respectively. It was found that the level of TREM2 was increased in PAMs that were expressing mCherry-tagged Nsp2 (Fig 2D). Myc-tagged TREM2 and ten mCherry-tagged NSPs were then co-transfected into HEK293T cells, respectively. We observed that Nsp2, Nsp3, Nsp5, and Nsp7 co-localized with TREM2 (Fig 2E). Likewise, we performed co-IP assays to screen for TREM2-interacting viral NSPs. We found that exogenous TREM2 co-precipitated with Nsp2 in HEK293T cells (Fig 2F), while no interaction between TREM2 and Nsp3, Nsp5, or Nsp7 was detected (S2B Fig). To further investigate the positive interaction between TREM2 and Nsp2, Myc-tagged TREM2 was overexpressed in Marc-145 cells, and then cells were infected with PRRSV. As expected, a Myc antibody immunoprecipitated PRRSV Nsp2 (Fig 2G). Moreover, PAMs were mock-infected or infected with PRRSV, and a TREM2 antibody immunoprecipitated PRRSV Nsp2 in PRRSV-infected cells (S2A Fig). To map the domain(s) required for the TREM2/Nsp2 interaction, we co-transfected mCherry-tagged Nsp2 and Myc-tagged full-length or truncated TREM2 into HEK293T cells. We found that TREM2 constructs comprising the cytoplasmic tail region co-precipitated with Nsp2, while TREM2 fragments lacking the cytoplasmic tail region had no interaction with Nsp2, indicating that the cytoplasmic tail domain might contain the binding region of viral Nsp2 (Fig 2H). To explore whether the TREM2/Nsp2 interaction affects TREM2 expression, we constructed two mCherry-tagged truncated Nsp2 (Nsp2-N and Nsp2-M) [46] and transfected them into PAMs (S2C Fig). We observed transcriptional upregulation of TREM2 in Nsp2-transfected cells and Nsp2-N-transfected cells, but there was no significant difference in TREM2 expression between Nsp2-M-transfected cells and control cells (S2D Fig). Furthermore, we co-transfected Myc-tagged TREM2 and mCherry-tagged Nsp2 or truncated Nsp2 into HEK293T cells and Marc-145 cells. The results revealed that the truncated Nsp2-N retained the ability to interact with TREM2, while the truncated Nsp2-M did not (S2E and S2F Fig), indicating that Nsp2-N (aa1-405) is responsible for the interaction with TREM2 and therefore affects TREM2 levels. Taken together, the cytoplasmic tail domain of TREM2 is required and sufficient for facilitating PRRSV replication via binding to PRRSV Nsp2.

### TREM2 downregulation activates downstream PI3K/Akt and ERK1/2 signaling

**Fig 3:**
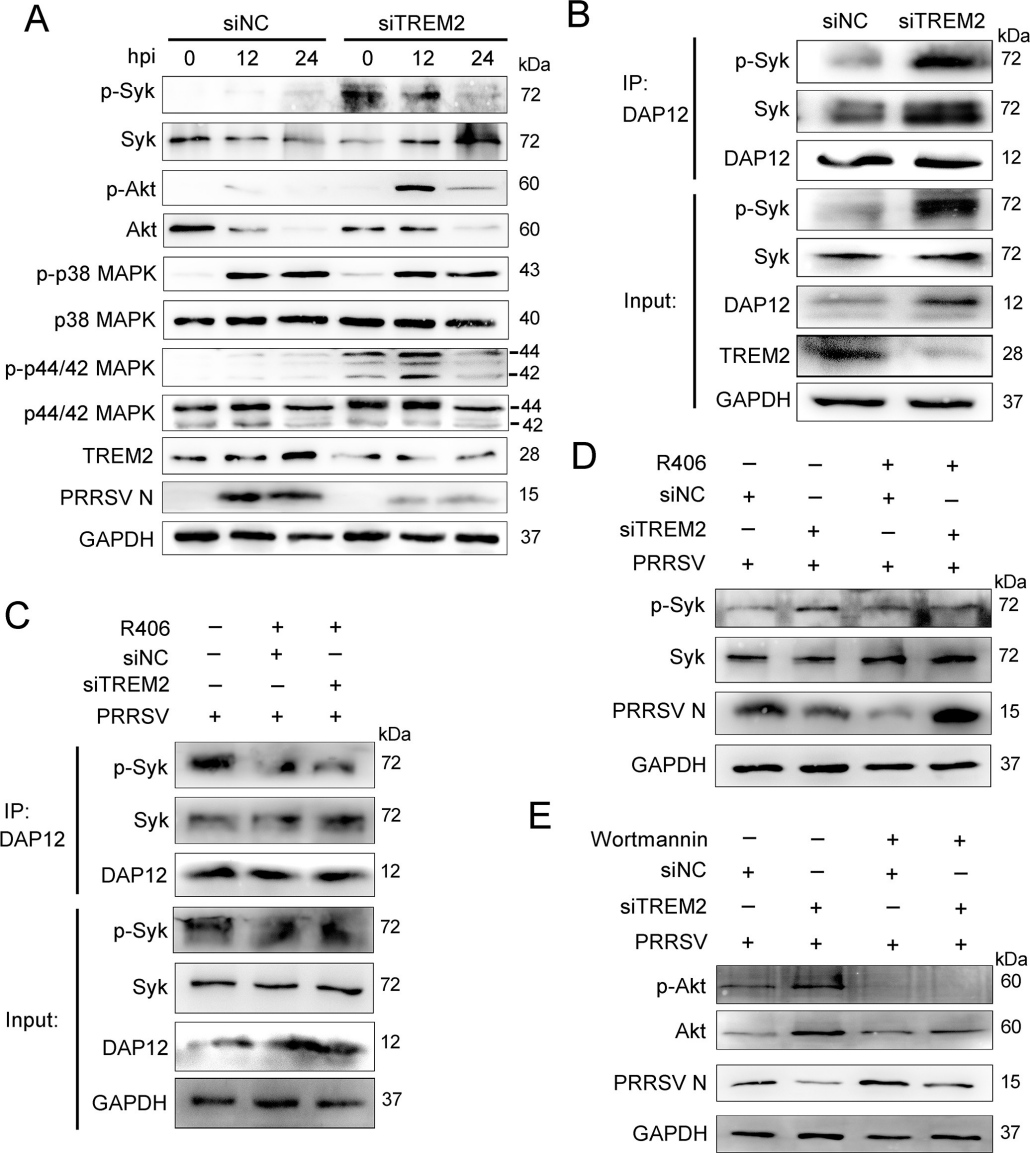
TREM2 knockdown promotes the recruitment of Syk and the activation of the PI3K and ERK1/2 pathways. (A) PAMs were infected with PRRSV (MOI = 1) for the indicated periods after TREM2 knockdown. Western blots shown the detection phosphorylated Sky, Akt, p38 MAPK, and p44/42 MAPK using specific antibodies. GAPDH is shown as an internal control. (B) PAMs were transfected with siNC or siTREM2-1 for 36 h, and cell lysates were immunoprecipitated with DAP12 antibody. Immunoblots of Syk, p-Syk, and DAP12 are shown. GAPDH is shown as an internal control. (C) PAMs with TREM2 knockdown were infected with PRRSV (MOI = 1) in the presence of R406 (Syk inhibitor, 5 μM). Cell lysates were immunoprecipitated with DAP12 antibody, and immunoblots are shown to depict the change in the DAP12-Syk association when treated with the Syk inhibitor. GAPDH is shown as an internal control. (D and E) TREM2-knockdown PAMs were infected with PRRSV (MOI = 1) in the presence or absence of R406 (D) or Wortmannin (PI3K/Akt inhibitor, 1 μM) (E) for 24 h. Western blot analysis was conducted to illustrate the protein levels of phosphorylated Syk, phosphorylated Akt, and PRRSV N, respectively. GAPDH is shown as an internal control. Data are representative of the results of three independent experiments.

TREM2 signaling relies on DAP12, a signaling adaptor containing an ITAM. The phosphorylated ITAM recruits and activates spleen tyrosine kinase (Syk), leading to a cellular immune response [24]. To identify the activated signaling driven by TREM2 following PRRSV infection, PAMs were transfected with TREM2 siRNA or negative siRNA for 24 h and then infected with PRRSV. As shown in Fig 3A, TREM2 knockdown catalyzed the increased phosphorylation of Syk, Akt, and ERK1/2 in PAMs compared to the negative control. Immunoprecipitation assays indicated that DAP12 constitutively interacted with Syk and phosphorylated Syk, and this interaction was enhanced in PAMs with TREM2 knockdown (Fig 3B). The Syk inhibitor R406 and PI3K inhibitor Wortmannin were used to inhibit the phosphorylation of Syk and Akt, respectively. R406 significantly decreased the phosphorylation of Syk and the combination between phosphorylated Syk and DAP12 during PRRSV infection (Fig 3C). Interestingly, in TREM2 knockdown cells treated with inhibitors, the PRRSV N protein was increasingly rescued compared to those not treated with an inhibitor (Fig 3D and 3E). These data illustrate that TREM2 silencing provokes the recruitment of Syk and the activation of the PI3K and ERK1/2 pathways, which are associated with PRRSV replication.

### TREM2 knockdown leads to early activation of the TLR4-NF-κB pathway during PRRSV infection

**Fig 4:**
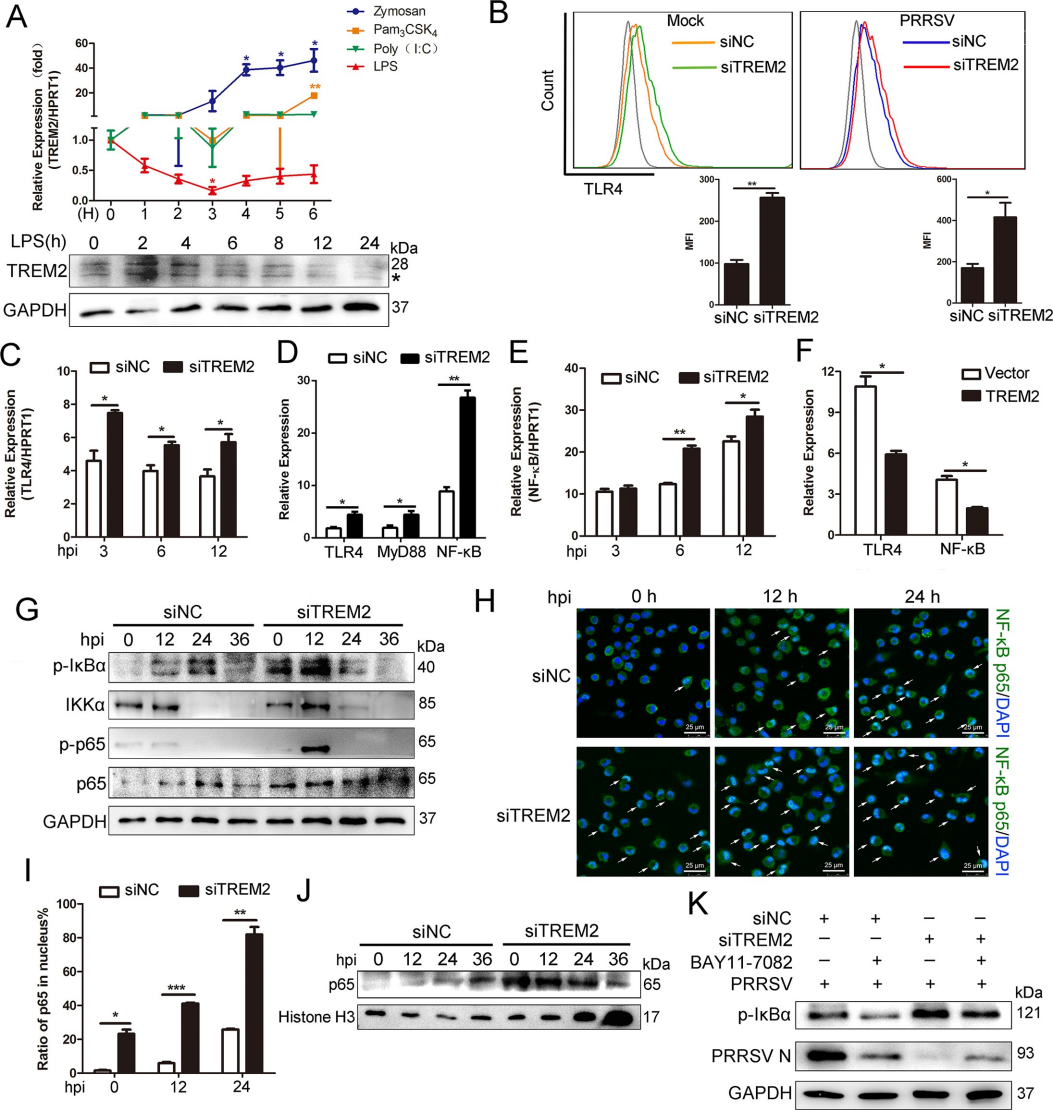
TREM2 knockdown accelerates the activation of the TLR4-NF-κB pathway. (A) PAMs were treated with different TLR agonists (40 particles Zymosan, 2 μg/mL Pam_3_CSK_4_, 100 ng/mL LPS and 10 μg/mL Poly (I:C)) and the transcription levels of TREM2 was examined by qRT-PCR. Simultaneously, the expression of TREM2 in PAMs upon LPS stimulation for the indicated periods is shown, as detected by western blot analysis. GAPDH is shown as an internal control. Asterisks marked the bands of glycosylated TREM2. (B) Representative histograms for flow cytometry analysis of cell surface TLR4 immunostaining for PAMs with the indicated conditions. The mean fluorescence intensity was quantified based on the flow cytometry analysis above. (C) After silencing TREM2 for 24 h, PAMs were infected with PRRSV (MOI = 1). Gene expression of TLR4 was detected by qRT-PCR, at the early stage of viral infection. (D) PAMs were transfected with siNC or siTREM2-1 for 24 h, qRT-PCR was performed to depict the mRNA abundance of TLR4, MyD88, and NF-κB. (E) Gene expression of NF-κB is shown, as detected by qRT-PCR, at the early stage of infection. (F) PAMs were transfected with pcDNA3.1-control (vector) or pcDNA3.1-TREM2 for 24 h, and infected with PRRSV (MOI = 1) for 24 h. qRT-PCR is shown to depict levels of TLR4 and NF-κB mRNA. (G) PAMs were infected with PRRSV (MOI = 1) for the indicated periods after transfection with siNC or siTREM2-1 for 24 h. Western blots are shown to detect phosphorylated IκBα, NF-κB p65, and IKKα. GAPDH is shown as an internal control. (H) Immunofluorescence analysis of the nuclear translocation of NF-κB p65 and the co-localization between NF-κB p65 (green) and cell nucleus (blue) (Bar, 25 μm). (I) Quantification of NF-κB p65 translocation based on the visual images in H. ~300 cells for each time point. (J) PAMs were treated as described in G, cells were collected, and a cell fractionation assay was performed. Western blots are shown to detect NF-κB p65. Histone H3 is used as a nuclear internal control. (K) PAMs with TREM2 knockdown were infected with PRRSV (MOI = 1) in the presence or absence of BAY11-7082 (NF-κB inhibitor, 10 μM) and western blots are shown to detect protein levels of phosphorylated IκBα and PRRSV N. GAPDH is shown as an internal control. Data are representative of the results of three independent experiments (mean ± SE). Significant differences compared to the control group are denoted by * (*P* < .05), ** (*P* < .01) and *** (*P* < .001).

Previous studies have shown that the expression of TREM2 on the cell surface is regulated by TLR stimulation and TREM2 is downregulated upon LPS challenge in peritoneal macrophages [47]. TREM2 plays a role in fine-tuning innate responses by dampening signals induced by TLRs [29, 30]. We speculated TREM2 controlled the immune response against PRRSV infection through TLRs and their signaling. To address this hypothesis, we used TLR agonists Zymosan, Pam_3_CSK_4_, LPS, and poly (I:C) to stimulate PAMs and then detected the expression of TREM2. As shown in Fig 4A, Zymosan, Pam_3_CSK_4_, and poly (I:C) promoted the transcription of TREM2. Surprisingly, TREM2 expression gradually decreased when PAMs were stimulated with LPS, a specific TLR4 agonist, indicating that TLR4 is involved in TREM2 signaling. Next, we detected TLR4 expression on the cell membrane using flow cytometry when TREM2 was knocked down. Silencing of TREM2 boosted the expression of membrane-associated TLR4 in virus-infected or mock-infected cells (Fig 4B). Meanwhile, TREM2 knockdown enhanced the mRNA expression of TLR4 at the early stage of infection (Fig 4C). Furthermore, we determined whether TREM2 activates signaling downstream of the TLR4 pathway, such as NF-κB signaling. We conducted a TREM2 interference assay and found that TREM2 knockdown increased the transcription of TLR4, MyD88, and NF-κB (Fig 4D). Additionally, NF-κB mRNA was induced as early as 6 hpi in TREM2-silenced PAMs and maintained a high transcription level throughout the early stage of infection (Fig 4E). In contrast, TREM2 overexpression decreased the transcription of TLR4 and NF-κB induced by PRRSV (Fig 4F). Western blot analysis revealed that phosphorylation of IκBα and p65 occurred much earlier (0 hpi) in TREM2-silenced PAMs compared to control PAMs. Moreover, the phosphorylation of IκBα and p65 was clearly increased and reached a peak at 12 hpi in TREM2-silenced PAMs (Fig 4G). Immunofluorescence analysis of the nuclear translocation of p65, which represents the activation of NF-κB, was conducted. Compared with the negative control, translocation of p65 (green) into the nucleus of infected cells occurred much earlier following TREM2 silencing and reached a maximum (~ 80%) at 24 hpi (Fig 4H and 4I). Accordingly, we performed a cell fractionation assay and found that nuclear accumulation of endogenous p65, measured following infection, occurred at earlier time points and to a greater magnitude in TREM2-silenced PAMs, as compared with controls (Fig 4J). A rescue experiment was implemented using BAY11-7082, an NF-κB inhibitor, which partially rescued the replication of PRRSV following TREM2 knockdown treatment (Fig 4K). Taken together, TREM2 knockdown dramatically accelerates the activation of NF-κB induced by PRRSV.

### TREM2 knockdown accelerates the proinflammatory response and increases type I interferon production

**Fig 5:**
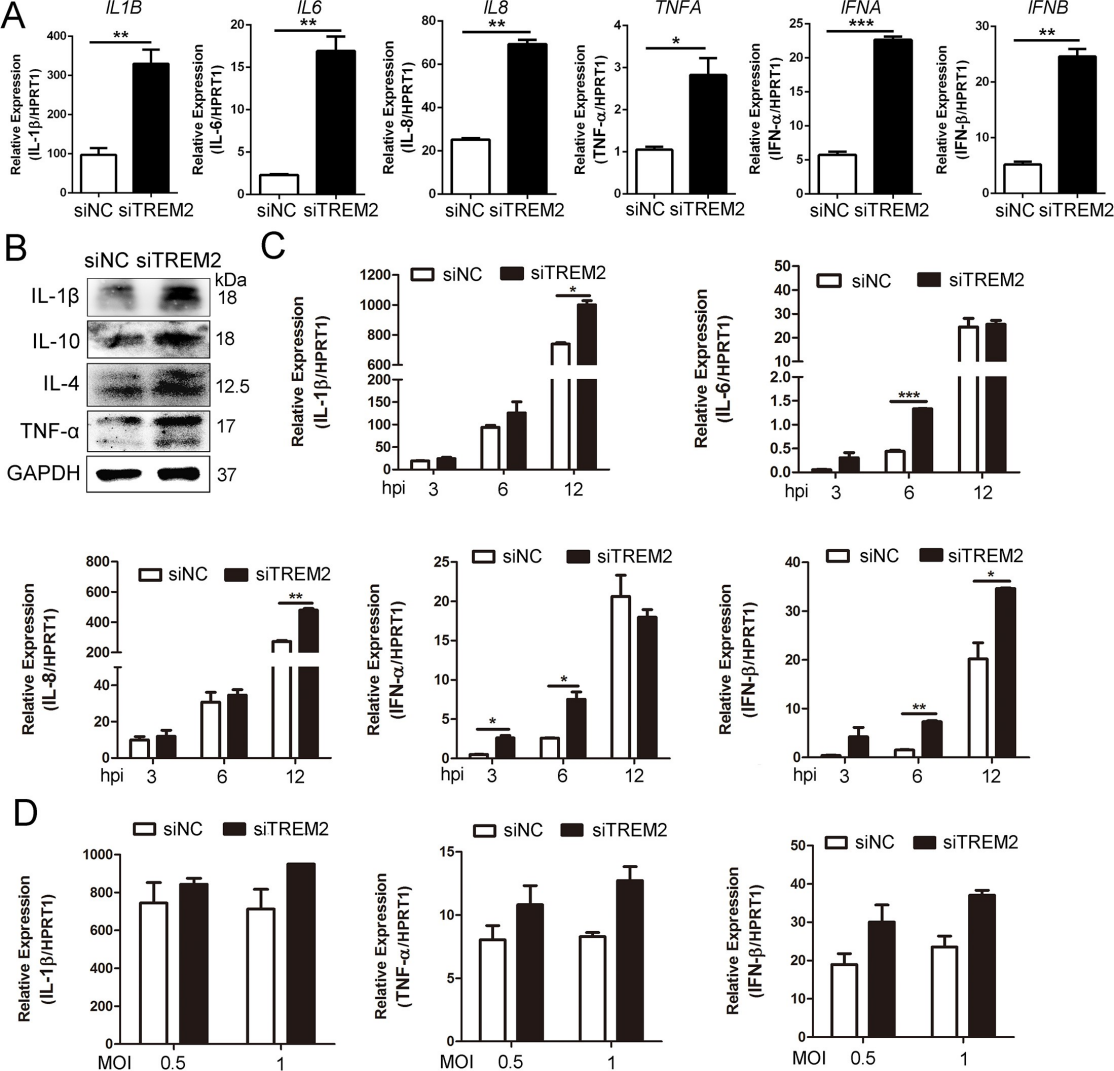
Silencing of TREM2 increases the production of inflammatory cytokines and type I interferons. (A) PAMs were transfected with siNC or siTREM2-1 for 24 h, gene expression of IL-1β, IL-6, IL-8, TNF-α, IFN-α, and IFN-β are shown using qRT-PCR. (B) Protein expression of IL-1β, IL-10, IL-4, and TNF-α are shown, as detected by western blot analysis in the TREM2 knockdown assay. GAPDH is shown as an internal control. (C) PAMs were transfected with siNC or siTREM2-1 for 24 h and infected with PPRSV (MOI = 1). Cells were collected at the indicated periods. Proinflammatory cytokine (IL-1β, IL-6, and IL-8) and type I interferons (IFN-α and IFN-β) mRNA expression is shown, as detected by qRT-PCR. (D) PAMs were treated as described in A and infected with PRRSV at different MOIs (0.5 or 1), transcription of IL-1β, TNF-α, and IFN-β is shown, as measured by qRT-PCR. Data are representative of the results of three independent experiments (mean ± SE). Significant differences compared to the control group are denoted by * (*P* < .05), ** (*P* < .01) and *** (*P* < .001).

TREM2 is involved in regulation of the virus-triggered inflammatory response [38]. To summarize the above findings, NF-κB signaling was activated at early time points following RNA interference of TREM2, prior to PRRSV-induced NF-κB activation. Accordingly, we investigated whether TREM2 regulates the production of PRRSV-induced inflammatory cytokines and type I interferons in PAMs. As shown in Fig 5A, PAMs with a TREM2 knockdown produced more (about 3~5 fold) inflammatory cytokines (IL-1β, IL-6, IL-8, and TNF-α) and type I interferons (IFN-α and IFN-β) than those with the negative control. The protein levels of IL-1β, IL-4, IL-10, and TNF-α were significantly increased in TREM2-silenced PAMs (Fig 5B). Next, after PAMs were infected with PRRSV following TREM2 knockdown, we detected the transcription of proinflammatory cytokines at the early stage of infection. Proinflammatory cytokines and type I interferons were clearly induced as early as 3 hpi and were significantly augmented from 6–12 hpi in TREM2-silenced PAMs (Fig 5C). TREM2 knockdown also boosted PRRSV-induced cytokine (IL-1β, TNF-α, IFN-β) transcription at different MOIs (Fig 5D). These results illustrate that TREM2 knockdown potentiates the transcription of proinflammatory cytokines and type I interferons at the early stage of PRRSV infection.

### TREM2 increases the expression of CD163 and decreases the cleavage of membrane CD163 mediated by ADAM17

**Fig 6:**
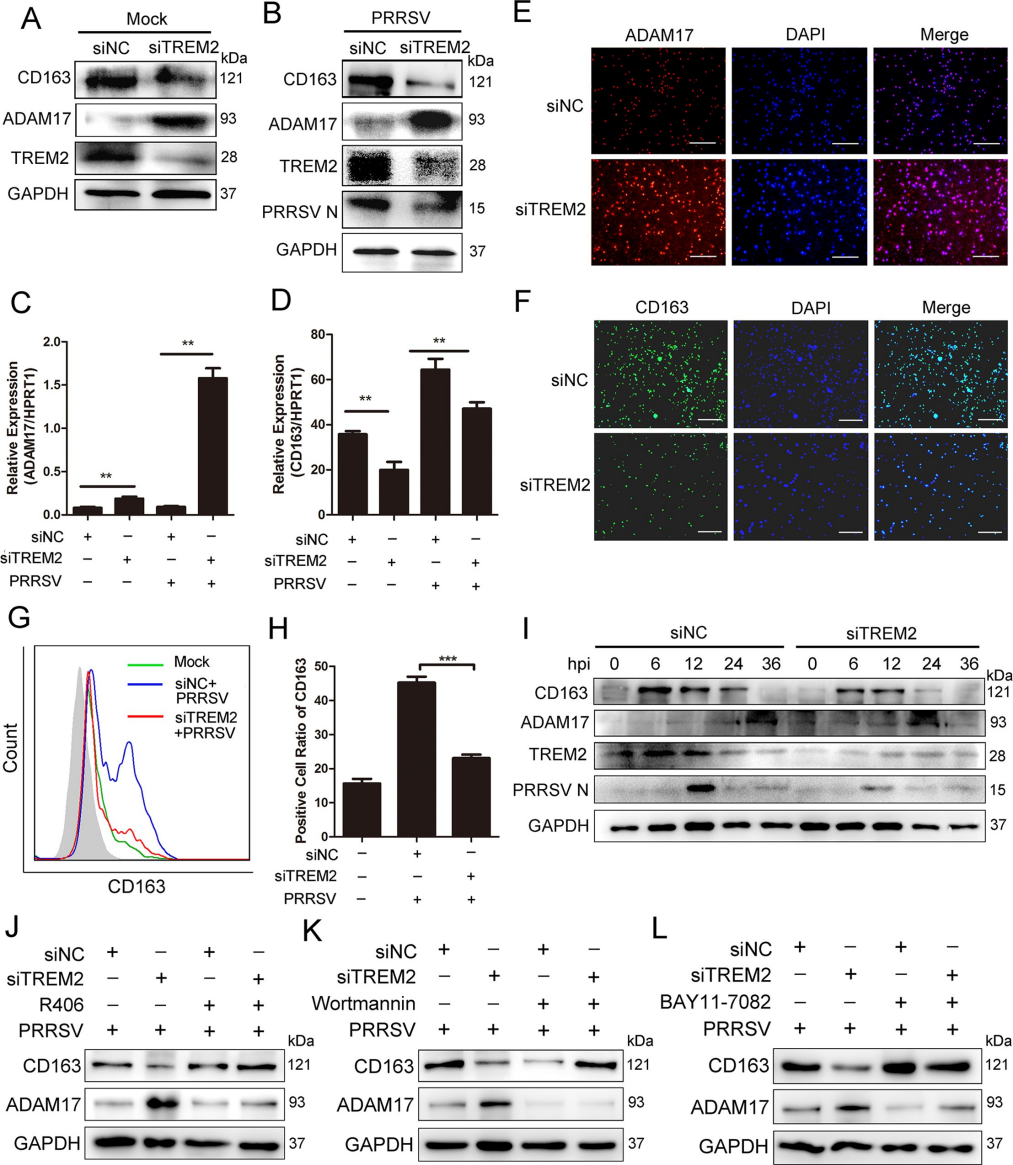
TREM2 regulates the expression of ADAM17 and CD163. (A) PAMs were transfected with siNC or siTREM2-1 for 24 h, and cells were collected. The protein levels of CD163 and ADAM17 are shown, as detected by western blot. GAPDH is shown as an internal control. (B) PAMs were transfected with siNC or siTREM2-1 for 12 h and infected with PRRSV (MOI = 1) for an additional 24 h. The CD163, ADAM17 and PRRSV N protein expression is shown, as measured by western blot. GAPDH is shown as an internal control. (C and D) PAMs were transfected with siNC or siTREM2-1 for 24 h, and then mock-infected or infected with PRRSV (MOI = 1) for 24 h. Expression of ADAM17 (C) and CD163 (D) is shown, as measured using qRT-PCR. (E and F) Immunofluorescence analysis is shown to detect the expression of ADAM17 (E) and CD163 (F) after down-regulating TREM2 expression (Bar, 200 μm). (G) Representative histograms for flow cytometry analysis of cell surface CD163 immunostaining for PAMs with the indicated conditions. (H) Corresponding positive cell ratio of cell surface CD163 based on analysis conditions in G. (I) PAMs with TREM2 knockdown were infected with PRRSV (MOI = 1) for the indicated periods. Cells were collected and protein levels of CD163, ADAM17, TREM2, and PRRSV N are shown using western blot analysis. GAPDH is shown as an internal control. (J—L) PAMs with TREM2 knockdown were infected with PRRSV (MOI = 1) in the presence or absence of R406 (5 μM) (J), Wortmannin (1 μM) (K) or BAY11-7082 (10 μM) (L) and western blots are shown to detect protein levels of ADAM17 and CD163. GAPDH is shown as an internal control. Data are representative of the results of three independent experiments (mean ± SE). Significant differences compared to the control group are denoted by * (*P* < .05), ** (*P* < .01) and *** (*P* < .001).

CD163 is a key factor in the initiation of PRRSV infection. It is regulated by many proinflammatory cytokines and is cleaved by ADAM17 [18, 48]. As previously covered, TREM2 silencing resulted in an early proinflammatory cytokine and type I interferon response. We investigated whether TREM2 regulates the expression of ADAM17 and CD163 via proinflammatory cytokines. Therefore, we used siRNA to deplete cells of TREM2 and detected the expression of ADAM17 and CD163. As expected, TREM2 knockdown clearly enhanced the protein level of ADAM17, while decreasing the expression of CD163 following PRRSV infection (Fig 6A and 6B). ADAM17 mRNA levels were significantly increased, but CD163 mRNA levels showed a significant reduction in each case when compared to the levels in PAMs transfected with negative siRNA (Fig 6C and 6D). Similarly, an immunofluorescence assay revealed that the expression of ADAM17 was increased (Fig 6E), whereas the fluorescence intensity of the CD163 protein was obviously reduced in TREM2-silenced PAMs following PRRSV infection (Fig 6F). Since activated ADAM17 cleaves the ectodomain of CD163, which mediates CD163 shedding from the cell membrane and the production of sCD163, we detected membrane CD163 expression and sCD163 production following the RNA interference assay. As expected, TREM2 knockdown also induced a significant reduction of membrane-associated CD163 in PAMs based on flow cytometry (Fig 6G and 6H). Consistently, sCD163 production was higher in the supernatant of TREM2-silenced PAMs at 12, 24, 36 hpi when compared with the controls (S3A Fig). Fig 6I further demonstrates that TREM2 knockdown induced a reduction of CD163 in PAMs following PRRSV infection and ADAM17 expression occurred as early as 0 hpi and to a greater magnitude at 24 hpi in TREM2-silenced PAMs, compared to the negative control. On the contrary, TREM2 overexpression triggered a significant increase of CD163 expression (S3B Fig) and membrane-localized CD163 (S3C and S3D Fig), while decreasing the expression of ADAM17 (S3B Fig). These data indicate that the regulatory effect of TREM2 on PRRSV infection is achieved by fine-tuning the expression of ADAM17 and CD163.

To demonstrate that TREM2 knockdown reduces cytokine-mediated CD163 expression to inhibit virus infection via Syk/PI3K and TLR4/NF-κB signaling, we first explored whether TREM2 knockdown promotes the expression of proinflammatory cytokines and type I interferons via these pathways. PAMs were mock-treated or treated with Syk inhibitor R406, PI3K inhibitor Wortmannin, or NF-κB inhibitor BAY11-7082 in conditions of TREM2 knockdown and were then mock-infected or infected with PRRSV. We found that the mRNA expression of IL-1β (S4A Fig), IL-8 (S4B Fig), TNF-α (S4C Fig), IFN-β (S4D Fig), IFN-α (S4E Fig). and NF-κB (S4F Fig) was upregulated when TREM2 was silenced. However, this upregulation mediated by TREM2 silencing was significantly decreased when cells were exposed to inhibitors in the presence or absence of PRRSV, suggesting that TREM2 silencing intensifies the expression of cytokines via Syk/PI3K and NF-κB signaling, which may affect the expression of CD163 [15, 49]. To understand whether TREM2 regulates ADAM17 and CD163 expression through Syk/PI3K and NF-κB signaling, we next detected the expression of ADAM17 and CD163. As expected, the upregulation of ADAM17 induced by TREM2 knockdown was significantly decreased when cells were treated with inhibitors (Fig 6J, 6K and 6L and S4G Fig). Consistently, the protein and mRNA expression of CD163 was decreased in TREM2-silenced PAMs without inhibitor treatment. However, the TREM2-silencing-mediated inhibition of CD163 was clearly rescued when Syk, PI3K, or NF-κB signaling was blocked (Fig 6J, 6K and 6L and S4H Fig). These results suggest that silencing of TREM2 upregulates ADAM17 and downregulates CD163 via Syk/PI3K and NF-κB signaling. To further investigate whether CD163 downregulation triggered through TREM2 silencing is dependent on cytokines, we used TLR4 inhibitor TAK-242 or dexamethasone (an anti-inflammatory drug) [50] to treat TREM2 knockdown cells. We found that the decreased CD163 expression was rescued in TREM2 knockdown cells treated with inhibitor or dexamethasone compared to the cells without treatment (S4I, S4J and S4K Fig), indicating that the effect of TREM2 on CD163 is cytokine-mediated. Inhibitors partially rescued PRRSV replication in TREM2 knockdown cells, since CD163 expression was rescued (S4J, S4K, and S4L Fig). Taken together, these data demonstrate that TREM2 knockdown decreases cytokine-mediated CD163 expression to inhibit virus infection via Syk/PI3K and TLR4/NF-κB signaling.

### Exogenous sTREM2 inhibition of PRRSV infection might be attributed to its competitive binding with viral envelope proteins

**Fig 7:**
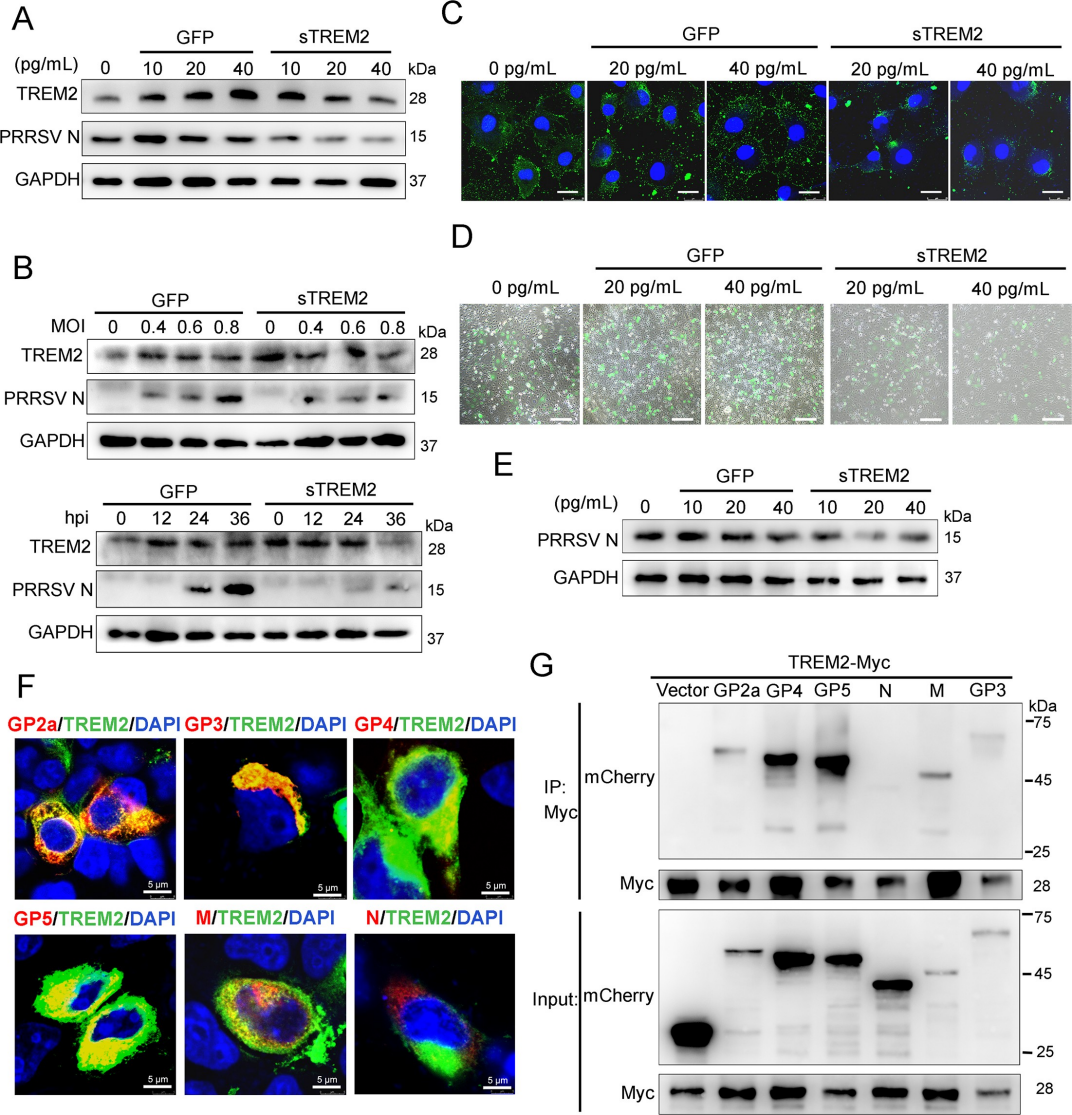
sTREM2 inhibits PRRSV replication by blocking viral attachment. (A) PAMs were infected with PRRSV (MOI = 1) in the presence or absence of different concentrations of sTREM2 or GFP protein (10, 20, 40 pg/mL) for 24 h. Purified GFP protein serves as a negative control. PRRSV N and endogenous TREM2 protein were detected by western blot. GAPDH is shown as an internal control. (B) Western blot was used to analyze the viral N protein and endogenous TREM2 levels in PAMs at different PRRSV MOIs or different infection periods in the presence or absence of sTREM2. Purified GFP protein serves as a negative control. (C) Marc-145 cells were incubated with PRRSV (MOI = 5) in the presence or absence of different concentrations of sTREM2 for 3 h at 4°C. Immunofluorescence analysis of PRRSV (green) attachment to the cell surface (Bar, 25 μm) was assessed. Purified GFP protein serves as a negative control. (D and E) After incubation at 4°C for 3 h, unbound viral particles were removed and cells were switched to 37°C for 36 h. PRRSV was observed using fluorescence microscopy (Bar, 200 μm) (D). Western blot was used to detect the expression of PRRSV N. Purified GFP protein serves as a negative control (E). (F) Myc-tagged TREM2 and mCherry-tagged structural proteins (GP2a, GP3, GP4, GP5, M, and N) were co-transfected into HEK293T for 24 h, respectively. Immunofluorescence analysis of the co-localization between TREM2 (green) and the structural proteins (red) (Bar, 5 μm) was performed. (G) Likewise, HEK293T cells were transfected with Myc-tagged TREM2 and these mCherry-tagged structural proteins. Cell lysates were immunoprecipitated with a Myc antibody. Immunoprecipitated proteins were subjected to immunoblot with the mCherry antibody. Asterisks mark the expressed Myc-fusion proteins of TREM2 fragments. Data are representative of the results of three independent experiments.

sTREM2, an important form of TREM2, is generated via cleavage by ADAM10 and ADAM17, which plays a role in promoting an inflammatory response and macrophage survival [38, 51, 52]. To identify the potential function of sTREM2 during PRRSV infection, we first determined whether there is production of sTREM2 in cell supernatants following PRRSV challenge. We found a marked increase of sTREM2 in supernatants at different MOIs and time points after PRRSV infection (S5A and S5B Fig). Subsequently, sTREM2 and GFP protein were expressed and purified in *Escherichia coli* BL21 cells (DE3) (S5C and S5D Fig). Purified GFP protein served as a negative control. The PRRSV N protein was reduced in the presence of different concentrations of sTREM2 (Fig 7A). Meanwhile, sTREM2 showed potent antagonism against PRRSV in a dose- and time-dependent manner in PAMs (Fig 7B). The endogenous TREM2 expression in cells treated with sTREM2 was comparable to that in GFP protein-treated cells at 0, 12, and 24 hpi or different MOIs (Fig 7A and 7B), suggesting that the virus inhibition is indeed attributed to the effect of sTREM2. To identify which stage of viral infection was blocked by sTREM2, we carried out viral attachment and entry assays. The results revealed that sTREM2 reduced virus particles attaching to the cell surface (Fig 7C, 7D and 7E), but had no influence on the entry of the virus particles in Marc-145 cells, suggesting that sTREM2 restrains PRRSV infection by blocking viral attachment (S5E Fig). Given that TREM2 is a transmembrane glycoprotein (GP), we speculated that there was an interaction between sTREM2 and viral structural proteins. To test this hypothesis, Myc-tagged TREM2 and different mCherry-tagged viral structural proteins were co-transfected into HEK293T cells. Immunofluorescence assays showed that TREM2 co-localized with glycoprotein (GP) 2a, GP3, GP4, GP5, and M protein, but not with N protein (Fig 7F). IP assays further showed that TREM2 interacted with PRRSV GP2a, GP3, GP4, GP5, and M protein, but there was no interaction between TREM2 and N protein (Fig 7G). To screen the domain(s) of TREM2 required for its interaction with viral structural proteins, we selected GP4 and GP5, which were largely precipitated by TREM2. To conduct IP assays. Myc-tagged full-length or truncated TREM2 and mCherry-tagged GP4 or GP5 were co-transfected into HEK293T. We found that TREM2 constructs comprising the Ig-like V-type domain co-precipitated GP4 or GP5, suggesting that an Ig-like V-type domain of TREM2 might be the binding region of viral GP4 and GP5 (S5F and S5G Fig). Collectively, sTREM2 plays a role in protection against PRRSV infection.

### TREM2 expression is upregulated post PRRSV infection in piglets

**Fig 8:**
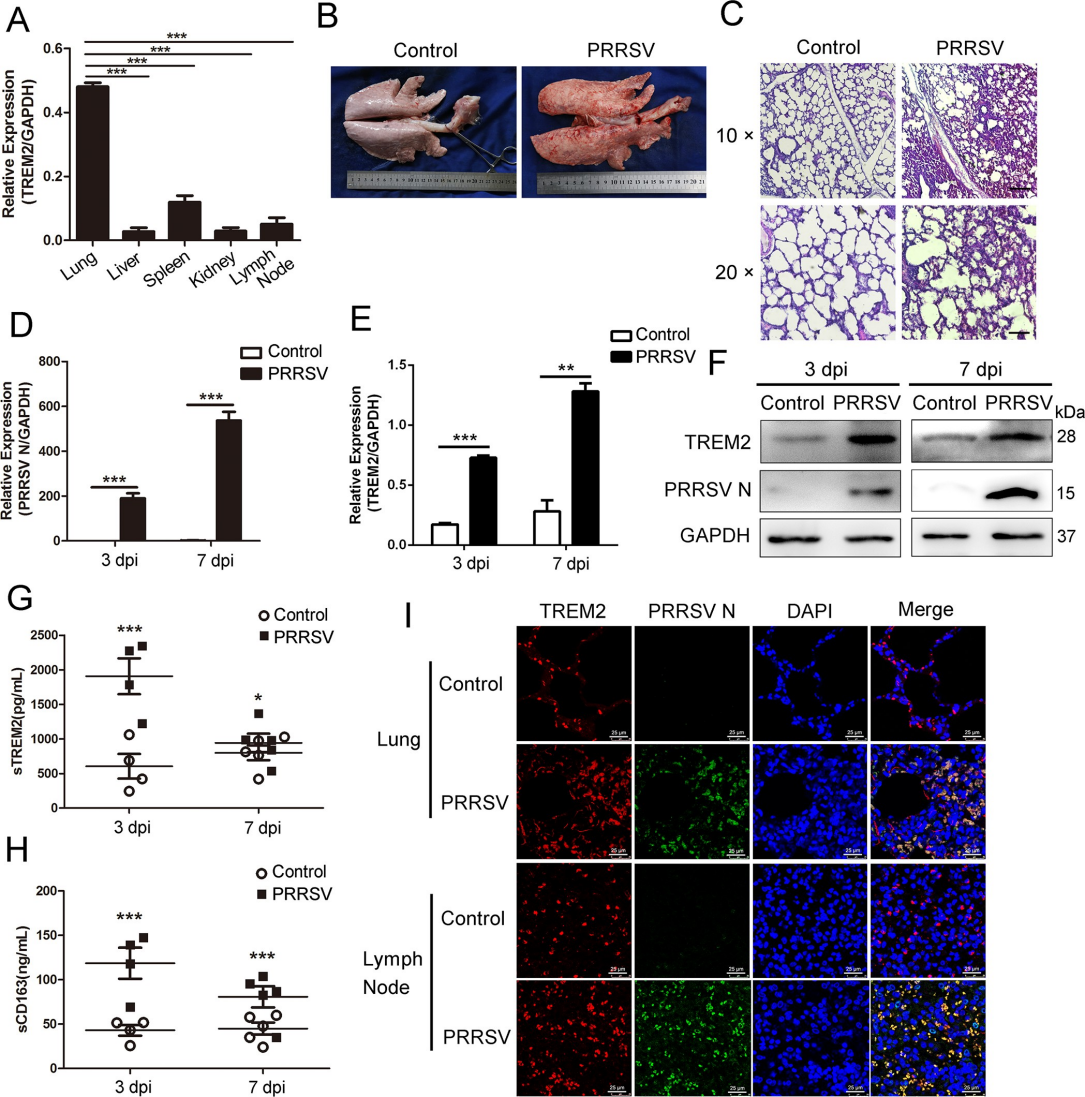
Piglets with PRRSV infection show a higher expression of TREM2. Twenty piglets were divided into two groups, 10 piglets per group, and kept in the same conditions. One group of piglets was challenged with the HP-PRRSV Li10 strain using nasal drip, the other group was injected with PBS. On days 3 and 7 post-challenge, we selected five piglets from each group for the following experiment. (A) Different tissues were separated from uninfected piglets, and the transcription of TREM2 in different tissues is shown, as measured by qRT-PCR. (B) Pictures are shown of the dorsal side of lungs from healthy control animals and animals challenged with PRRSV on day 7 post-challenge. (C) Lung paraffin sections from control and PRRSV infected piglets on 7 dpi were stained with H&E (Bar, 200 μm or 100 μm). (D and E) Lungs were separated on days 3 and 7 post-infection, and qRT-PCR was used to detect the mRNA expression of PRRSV ORF7 (D) and TREM2 (E) in PBS controls and PRRSV infected animals. (F) The protein levels of PRRSV N and TREM2 in lungs were also analyzed by western blot. (G and H) Blood samples were collected on days 3 and 7 post-infection, and the detection of sTREM2 (G) and sCD163 (H) is shown, as measured by ELISA in PBS controls and PRRSV infected animals. (I) Lungs and lymph nodes paraffin sections from control and PRRSV infected piglets on 7 dpi were taken for immunofluorescent staining. TREM2 (red), PRRSV N (green), and DAPI (blue) were detected by laser scanning confocal microscopy (Bar, 25 μm). Data are representative of the results of three independent experiments (mean ± SE). Significant differences are indicated as follows: * (*P* < .05), ** (*P* < .01) and *** (*P* < .001).

To further validate the above results, we performed a PRRSV challenge *in vivo*. First, we detected the expression of TREM2 in different tissues and found that TREM2 was largely expressed in the lungs, which are the main target of PRRSV (Fig 8A). Based on the pathology assessed using visual examination and H&E staining, we found severe hemorrhage, congestion, and diffuse interstitial pneumonia occurred in virus-infected lungs (Fig 8B and 8C). qRT-PCR analysis demonstrated that TREM2 mRNA was remarkably induced in lungs of infected piglets on 3 and 7 dpi, consistent with PRRSV N expression (Fig 8D and 8E). Similarly, TREM2 protein level in infected pigs was significantly higher than that of controls on 3 and 7 dpi (Fig 8F). The results of immunofluorescence indicated that the expression of TREM2 was enhanced in infected lungs and lymph nodes compared to PBS controls. TREM2 (red) and PRRSV (green) specifically co-localized in PAMs, and their colocalization was increased after PRRSV infection (Fig 8I). Blood samples were collected on days 3 and 7 post-challenge for the detection of sTREM2 and sCD163. It was clear that the production of sTREM2 and sCD163 in infected piglets was significantly higher than in uninfected controls (Fig 8G and 8H). Collectively, TREM2 is associated with PRRSV infection *in vivo*, and PRRSV potentiates the expression of TREM2 in lungs and lymph codes, as well as the production of sTREM2 in plasma.

## Discussion

**Fig 9:**
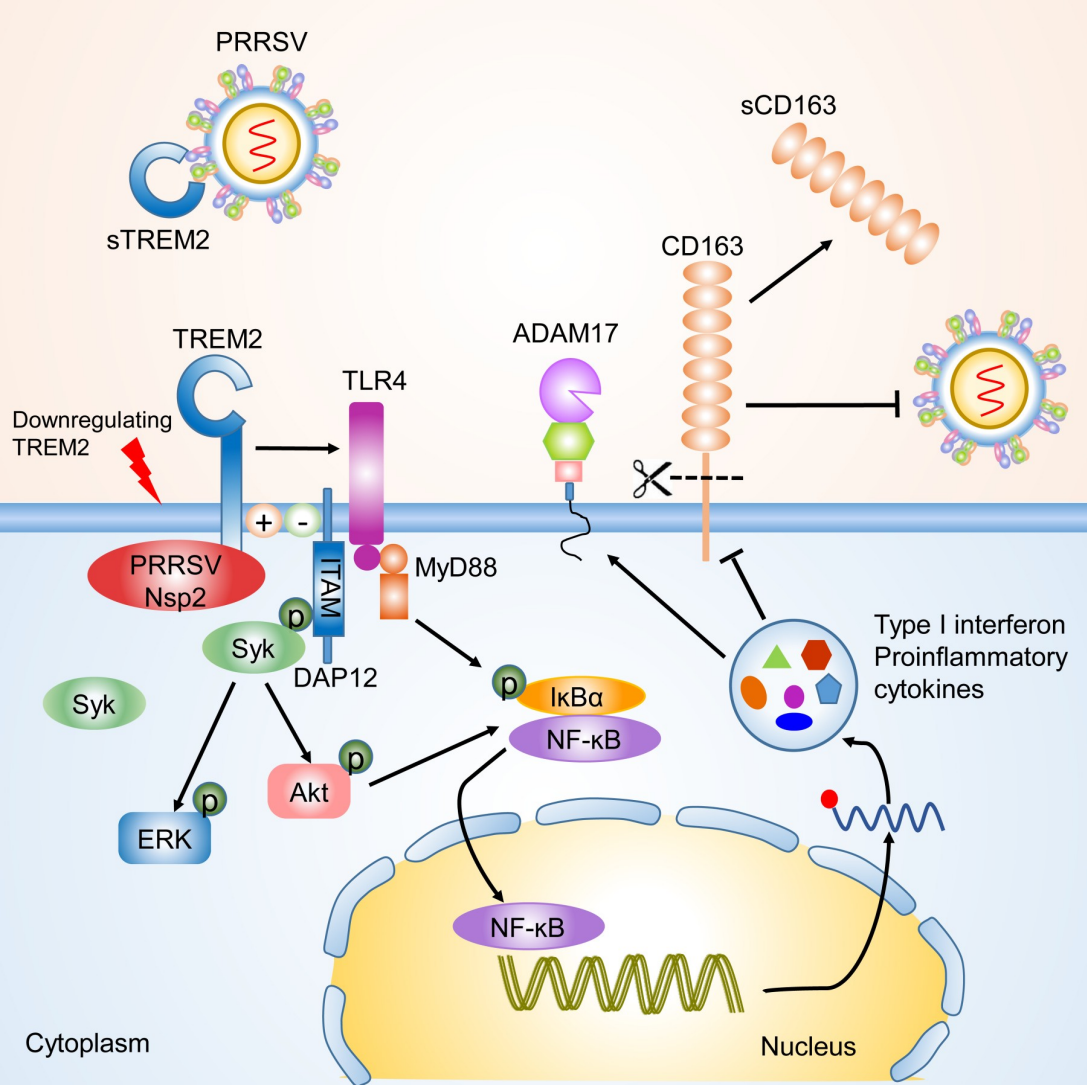
Schematic model of TREM2 regulation of PRRSV infection. TREM2 downregulation promotes the recruitment of Syk and the activation of downstream PI3K/Akt and ERK1/2 pathways. Simultaneously, TLR4-NF-κB signaling is triggered early in the viral infection, leading to an intensive and sustained proinflammatory response. Elevated proinflammatory cytokines and type I interferons reduce the expression of CD163 and increase the cleavage of CD163 mediated by ADAM17, which contributes to suppression of PRRSV infection. On the contrary, the cytoplasmic tail of TREM2 interacted with PRRSV Nsp2 to promote infection. Meanwhile, exogenous sTREM2 blocks PRRSV binding to cell receptors via competitively interacting with viral envelope proteins.

TREM2 is a novel immunoregulatory receptor with important functions within the anti-inflammatory response [22, 24, 27]. This report identified TREM2 as a regulatory factor for PRRSV infection. It particularly covers the following (Fig 9). First, the expression of TREM2 was increased with PRRSV challenge *in vivo* and *in vitro*. TREM2 promotes PRRSV infection via the interaction between its cytoplasmic region and PRRSV Nsp2. Second, silencing of TREM2 triggered downstream PI3K/AKT and TLR4-NF-κB signaling, which resulted in an early proinflammatory cytokine and type I interferon response. Third, increased proinflammatory cytokines in TREM2-silenced PAMs reduced CD163 expression and enhanced the shedding of sCD163 cleaved by ADAM17, leading to the inhibition of PRRSV infection. Finally, sTREM2 was upregulated upon PRRSV infection and exogenous sTREM2 inhibited PRRSV infection *in vitro*. Taken together, TREM2 functions as an immunoregulatory receptor in PRRSV infection.

The current study provides at least three novel insights regarding the role of TREM2 in PRRSV infection to be discussed. First, CD163 was not only indispensable for PRRSV infection, but was also associated with an inflammatory response [9, 53]. CD163 receptors are downregulated by various inflammatory factors, including IL-1α, IL-1β, IL-4, IL-8, LPS, TNF-α, IFN-γ, TLR4, etc. Under inflammatory conditions, ADAM17 is activated and rapidly cleaves CD163 from the surface of inflammatory macrophages [16]. Many studies have highlighted that inflammatory responses are regulated by the TREM2/DAP12 complex. Gao X reported that silencing of TREM2 enhances the inflammatory response of alveolar macrophages to LPS [54]. TREM2^-/-^ and DAP12^-/-^ mice were generated to show that the activation of macrophages produces high levels of inflammatory cytokines compared to WT mice [26]. In this study, TREM2 knockdown in PAMs intensified the PRRSV-induced proinflammatory response and amplified the release of proinflammatory cytokines and type I interferons, particularly at the early stage of infection (Fig 5). As expected, CD163 expression was downregulated, and the shedding of sCD163 mediated by ADAM17 was increased in TREM2-silenced PAMs (Fig 6). Early induction of proinflammatory cytokines and type I interferons in TREM2-silenced PAMs, coupled with the reduction of CD163, leads to the suppression of PRRSV (Fig 1D–1H).

Second, it was reported that NF-κB, ERK1/2 and p38 MAPK signaling participated in antiviral immunity following PRRSV infection [55–57]. There are dual roles of NF-κB regulation by PRRSV: inhibition and activation. It mainly manifests in the inhibition of the inflammatory response and type I interferon production in early infection while accelerating the production of proinflammatory cytokines later in the infection [58, 59]. In the current study, we demonstrated that TREM2 knockdown triggered the activation of NF-κB as early as 0 hpi in PAMs, prior to PRRSV-induced activation (Fig 4G–4K). The early activation of NF-κB leading to an intensive proinflammatory and type I interferon response is a possible means of inhibiting PRRSV infection. However, the NF-κB signaling pathway is reported to influence the expression of TREM1 and TREM2. More studies need to be performed to determine whether TREM2 expression is conversely regulated by the NF-κB pathway. TREM-2 interaction with TLRs plays a role in dampening immune responses. Studies showed that TREM2 or DAP12 deficient mice promote cytokine production by macrophages in response to LPS, zymosan, and CpG and suggested that TREM2 plays a role in reducing activation [26, 29]. Some studies reported that LPS reduces TREM2 expression and promotes the activation of cell surface markers [60]. In our study, zymosan and Pam_3_CSK_4_ upregulated TREM2 expression, while LPS downregulated TREM2 expression, which suggests TLR4 is involved in TREM2 knockdown signaling (Fig 4A).

Third, sTREM2 is an important form of TREM2, which is generated by the cleavage of ADAM10 and ADAM17 at the H157–S158 peptide bond [51, 52, 61]. sTREM2 was measured in plasma, cerebrospinal fluid, and bronchoalveolar lavage fluid in humans or animals suffering from bacterial, viral, and fungal infections. sTREM2 serves as an inflammatory marker with biological activity [62–65]. Here we found that the level of sTREM2 in serum dramatically increased at the early stage of infection (3–7 dpi) compared to controls *in vivo* (Fig 8G). Similarly, sTREM2 in cell supernatants also showed an increase during PRRSV infection *in vitro* (S5A and S5B Fig), indicating that an acute inflammatory response had occurred. Therefore, sTREM2 is a possible inflammatory biomarker in PRRSV infection. Interestingly, we observed that exogenous sTREM2 restrained the replication of PRRSV by blocking viral attachment (Fig 7C, 7D and 7E). We have no explanation for the higher level of TREM2 in GFP-treated cells than in the sTREM2-treated cells at 36 hpi (Fig 7B). Given that TREM2 is a transmembrane glycoprotein, we speculated that sTREM2 antagonized PRRSV binding to the cell surface via competitive binding with PRRSV structural proteins. As expected, IP assays indicated TREM2 had interactions with viral envelope proteins rather than the nucleocapsid protein (Fig 7F and 7G). Further investigation should be carried out to illustrate the binding mechanism between TREM2 and PRRSV. According to our results, sTREM2 inhibits PRRSV infection via blocking viral attachment *in vitro*, which shows potential therapeutic opportunities. However, sTREM2 has no effects on viral entry stage. Taking this into account, the antiviral effects of sTREM2 may work in the early stage of infection. Meanwhile, the dosage, administration route, and side effects should be taken into account when sTREM2 is tested in infected models *in vivo*. The emergence of sTREM2 in PRRSV-infected pigs suggests that it may serve as a potential diagnostic marker for disease prevention.

In summary, we demonstrated that knockdown of the TREM2 significantly accelerated the release of proinflammatory cytokines and type I interferons, which reduced the expression of CD163 and ultimately suppressed PRRSV infection. In addition, sTREM2 was increased upon PRRSV infection and exogenous sTREM2 inhibited PRRSV by blocking viral attachment. In general, this work highlights TREM2, a novel receptor that positively promotes PRRSV infection via an anti-inflammatory response, which provides a new perspective to cellular immune regulation following PRRSV infection as well as a new antiviral strategy.

## Materials and methods

### Ethics statement

All animal experiments described in this study have been performed on pigs. These piglets were kept in housing with suitable temperature and humidity where water and food were available ad libitum. The protocol license number was IACUC-DD-16-0901, which was approved by the Institutional Animal Care and Use Committee (IACUC) of Sun Yat-sen University. All animal work was carried out under the Laboratory Animals—Guideline of welfare and ethics written by the General Administration of Quality Supervision, Inspection and Quarantine of the People’s Republic of China.

### Cells and viruses

PAMs were isolated from 6-week-old specific-pathogen free piglets. Piglets were euthanized and the lungs were removed in a sterile environment. Sterile phosphate buffer solution (PBS) (Corning, USA) was lavaged into the lungs and gentle massage was used after lung lavage three times. Cells were collected by centrifugation for 10 min at 1000 rpm. After washing with RPMI-1640 medium (Gibco, USA), cells were frozen in 40% RPMI-1640 medium, 50% fetal bovine serum (FBS) (PAN, Germany), and 10% DMSO (Sigma, USA). Finally, cells were gradually frozen and stored in liquid nitrogen. PAMs were cultured in RPMI 1640 medium with 10% FBS at 37°C in 5% CO_2_. Marc-145 cell, derived from an African green monkey embryonic kidney cell, is an immortalized cell line which is permissive to PRRSV replication and commonly used in laboratories. Marc-145 cells and HEK-293T cells (China Center for Type Culture Collection, China) were maintained in Dulbecco’s modified Eagle’s medium (DMEM) (Corning, USA) containing 10% FBS. Two PRRSV strains, CHR6 (PRRSV-2, used for *in vitro* challenge assay) and Li10 (PRRSV-2, used for *in vivo* challenge assay) were provided by Dr. Heng Wang from South China Agricultural University. The two virus strains were propagated in Marc-145 cells and titrated to 50% tissue culture infective dose (TCID_50_).

### Challenge with PRRSV strain Li10 in pigs

Twenty piglets aged 4–6 weeks were selected for the viral challenge *in vivo*. Prior to infection, piglets were tested for both PRRSV antigen and antibody and were negative for both. Piglets were divided into two groups, 10 piglets per group. These piglets were kept in housing with suitable temperature and humidity where water and food were available ad libitum. After one week of acclimation, one group of piglets was challenged with the HP-PRRSV Li10 strain using nasal drip, and the other group was injected with PBS. The dose of intranasal injection was 2 × 10^5^ TCID_50_ Li10 in 3 mL culture medium. On days 3 and 7 post-challenge, we selected five piglets from each group. Selected piglets were euthanized, and different tissues were separated for tissue section, immunoblotting, and qRT-PCR. Blood samples were collected on the above-mentioned days for the detection of sTREM2 and soluble CD163 (sCD163).

### Antibodies and reagents

Western blot, immunoprecipitation, and immunofluorescence analysis were performed using the following antibodies: anti-PRRSV N (4A5) antibody (9041) was purchased from MEDIAN Diagnostics (MEDIAN, Republic of Korea). Anti-ADAM17 antibody (ab39163), anti-CD163 antibody (ab87099), anti-mCherry antibody (ab183628), and anti-TLR4 antibody (ab22048) were purchased from Abcam (Abcam, England). Anti-porcine IL-1β antibody (AF681), anti-porcine IL-4 antibody (MAB6543), anti-porcine TNF-α/TNFSF1A antibody (MAB6903), and anti-porcine IL-10 antibody (AF693) were purchased from R&D Systems (R&D, USA). TREM2 (D8I4C) rabbit mAb, (91068), phospho-Syk (Tyr525/526) (C87C1) rabbit mAb (2710), DAP12 (D7G1X) rabbit mAb (12492), GAPDH (14C10) rabbit mAb (2118), Syk (D3Z1E) rabbit mAb (13198T), phospho-Akt (Ser473) (D9E) rabbit mAb (4060S), Akt antibody (9272), p38 MAPK (D13E1) rabbit mAb (8690), phospho-p38 MAPK (Thr180/Tyr182) (D3F9) rabbit mAb (4511), p44/42 MAPK (Erk1/2) (137F5) rabbit mAb (4695), phospho-p44/42 MAPK (Erk1/2) (Thr202/Tyr204) (D13.14.4E) rabbit mAb (4370), NF-κB p65 (D14E12) rabbit mAb (8242), phospho-NF-κB p65 (Ser536) (93H1) rabbit mAb (3033), phospho-IκBα (Ser32) (14D4) rabbit mAb (2859), IKKα antibody (2682), Myc-tag (9B11) mouse mAb (2276), and histone H3 (D1H2) rabbit mAb (4499) were all purchased from Cell Signaling Technology (CST, USA). Mouse anti-pig CD163: FITC mAb (MCA2311GA) was purchased from Bio-Rad Laboratories (Bio-Rad, USA). Anti-PRRSV Nsp2 antibody was provided by Dr. Hanchun Yang from China Agricultural University.

Signaling activators or inhibitors were used as follows: Zymosan A (Z4250, Sigma, USA), Pam3CSK4.3HCl (1700–100, BioVision, USA), LPS (L2630, Sigma, USA), Poly (I:C) (P9582, Sigma), BAY11-7082 (B5556, Sigma, USA), Syk inhibitor R406 (SC1022, Beyotime, China), and the PI3K/Akt inhibitor wortmannin (S1952, Beyotime, China). All drugs were prepared in DMSO or water according to the company’s instruction.

### RNA interference

All small interference RNAs (siRNAs) against TREM2 and siRNA-negative control (NC) were designed and synthesized by Invitrogen. PAMs were seeded in six-well plates at 2×10^6^ cells/well, adherent cells were transfected with the indicated siRNAs at a final concentration of 10 nM using Lipofectamine RNAiMAX (13778, Invitrogen, USA) according to the manufacturer’s instructions for 24 h. Cells were infected with PRRSV for the indicated hours, 24 h post-transfection. Indicated siRNAs are listed in S1 Table.

### Expression vector construction

The cDNAs encoding TREM2 were obtained from PAM cDNA and subcloned into the pcDNA3.1-Myc vector (MY1023, EK-Bioscience, China). Three fragments of TREM2, designated 1–186 (residues 1 to 186), 1–165 (residues 1 to 165), and 1–126 (residues 1 to 126), were subcloned into the pcDNA3.1-Myc vector with a C-terminal Myc tag, respectively. The PRRSV Nsp1-Nsp12, GP2a, GP3, GP4, GP5, E, N, and two fragments of Nsp2 (Nsp2-N, aa1-405; Nsp2-M, aa323-844) genes were amplified from the PRRSV CHR6 strain and cloned into vector pmCherry-N (632523, Takara, Japan) with a C-terminal mCherry tag.

### Protein expression and purification

The extracellular domain of TREM2 gene and GFP gene were cloned into the expression vector PET32a and expressed in *Escherichia coli* BL21 cells (DE3) (CD601, TransGen Biotech, China). sTREM2 and GFP protein were induced with 1 mM IPTG (9030, Takara, Japan) at 37°C with shaking at 220 rpm for 12 h. After centrifugation, the sediments were resuspended with equilibration buffer and bacteria were lysed using the Ultrasonic Cell Disruptor. Verified sTREM2 and GFP protein were purified with Ni-NTA agarose (R90101, Invitrogen, USA), according to the manufacturer’s instructions. The concentration of purified sTREM2 and GFP protein was determined using the BCA Protein Assay Kit (23227, Thermo Scientific, USA).

### Western Blot

Six-well plate cell samples (2 × 10^6^ cells/well) were lysed in cell lysis buffer (Beyotime, China) containing phenylmethylsulfonyl fluoride (PMSF) (Beyotime, China) and phosphatase inhibitors. Cell lysates were subjected to 10–15% sodium dodecyl sulfate-polyacrylamide gel electrophoresis (SDS-PAGE) and semi-dry transferred onto a polyvinyl difluoride (PVDF) membrane (Roche, USA). Membranes were blocked with 3% bovine serum albumin (BSA) (Ruishu, China) in TBST (20 mM Tris-HCl pH 8.0, 150 mM NaCl, 0.05% Tween 20) for 1 h at room temperature. After blocking, membranes were probed with the indicated primary antibodies overnight at 4°C. Membranes were incubated with the corresponding secondary antibodies for 1 h at 37°C. The indicated proteins were visualized using an enhanced chemiluminescence (ECL) reagent (Fdbio Science, China).

### Immunofluorescence assay

Twelve-well plate cell samples were fixed with paraformaldehyde (Ruishu, China) for 10 min and then immediately permeabilized by 0.5% Triton (Ruishu, China) for 15 min at room temperature. After rinsing with PBS three times, cells were blocked with 1% BSA for 30 min and incubated with the primary antibody overnight at 4°C. Cells were incubated with the secondary antibody for 1 h, followed by staining with 4’,6-Diamidino-2-phenylindole dihydrochloride (DAPI) (C1002, Beyotime, China) in PBS for an additional 5 min. Fluorescent images were acquired with an inverted fluorescence microscope (NIKON ECLIPSE Ti2-U, Japan) or a confocal laser scanning microscope (TCS-SP5, LEICA, Germany).

### Measurement of sTREM2 and sCD163

Serum was separated from blood samples and the supernatants were collected from cultured cells. These samples were used for the detection of sTREM2 and sCD163. The porcine TREM2 ELISA kit and porcine CD163 ELISA kit (JL30780 and JL27715, Laibio, China) were used to measure the absorbance (450 nm) according to the manufacturer’s instructions.

### Flow cytometry

PAMs were harvested and fixed with paraformaldehyde for 10 min. Then, cells were gently rinsed with PBS three times. PAMs were stained with antibodies in 1% BSA for 1 h at room temperature. After rinsing with PBS three times, cells were resuspended in PBS at a concentration of 1 × 10^6^ cells/mL. Data were collected on a FACSCalibur flow cytometer (BD Bioscience, USA), and analyses were performed using the FlowJo software.

### Quantitative real-time reverse-transcription polymerase chain reaction (qRT-PCR)

Total RNA was extracted from cultured cells using the TRIzol reagent (Magen, China). Total RNA (1 μg) was reverse transcribed into cDNA using the Reverse Transcription System (A3500, Promega, USA) in a 10 μL reaction volume following the manufacturer’s protocol. Oligo (dT) 15 primer (C110A, Promega, USA) and random primer (C118A, Promega, USA) were the reverse-transcription primers. Reverse-transcription products were amplified with 2×RealStar Green Power Mixture (GenStar, China) on a LightCycler 480 Real-Time PCR System (LC480, Roche, Switzerland). The primers used for qRT-PCR are listed in S2 Table. GAPDH or HPRT1 was used as a housekeeping gene. Relative expression of target genes was calculated using the 2^−ΔΔCt^ method and normalized with the mean Ct of GAPDH or HPRT1.

### Co-immunoprecipitation (Co-IP) and immunoblotting

Cells were lysed in radio immunoprecipitation buffer (RIPA) (Beyotime, China), supplemented with PMSF and phosphatase inhibitors (Beyotime, China). Immunoprecipitation was performed according to the manufacturer’s instructions of the Dynabeads Protein G Immunoprecipitation Kit (10007D, Invitrogen, USA), 3 μg of the indicated antibodies were first bound to 40 μL Dynabeads for 1 h at room temperature. Cell lysate protein (1 mg) was incubated with the Dynabeads-Ab complex for 1 h at room temperature. Dynabeads-Ab-Ag complexes were washed with Washing Buffer three times and retained in Elution Buffer. The precipitates and whole-cell lysates were subjected to 10–15% SDS–PAGE and potentially associated proteins were tested by western blot as stated above.

### Hematoxylin-Eosin (H&E) staining

When piglets were euthanized on the indicated day post PRRSV challenge, lungs and other tissues were isolated. Pictures were taken from the dorsal side of the lungs to evaluate pathology. Lungs and lymph nodes were cut into appropriate tissue blocks and fixed in 4% neutral buffered formalin (Ruishu, China) for 18 h. After dehydration and embedding in paraffin, tissue samples were cut into 5 μm thick sections and mounted onto glass slides. For histopathology, sections were stained with H&E (NJJCTECH, China) according to the manufacturer’s instructions.

### Statistical analysis

All experiments were performed with at least three independent replicates. Student’s t-test and one-way ANOVA were used to analyze the data. Statistical analysis was performed using SPSS 17.0 and GraphPad Prism 5.0. Shown are means and error bars represent SE in all graphs. *P* < 0.05 was considered to be significant.

## Supporting information

S1 Fig PRRSV upregulates TREM2 on the transcriptional level. (A and B) PAMs were infected with PRRSV (MOI = 1) for the indicated time periods (0, 6, 12, 24 and 36 hpi) (A) or at different MOIs (0, 0.2, 0.4, 0.8, 1.6 and 3.2) for 24 h (B), transcriptional levels of TREM2 are shown, as detected by qRT-PCR. Data are representative of the results of three independent experiments (mean ± SE).
(TIF)

S2 Fig Nsp2 interacts with TREM2. (A) PAMs were mock-infected or infected with PRRSV (MOI = 1) for 24 h. Cell lysates were immunoprecipitated with TREM2 antibody, and the immunoblots with Nsp2 and TREM2 antibodies are shown. GAPDH is shown as an internal control. (B) Myc-tagged TREM2 and mCherry-tagged Nsp3, Nsp5 or Nsp7 were co-transfected into HEK293T for 36 h. Cell lysates were immunoprecipitated with Myc or IgG antibodies and the immunoblots are shown with mCherry antibodies. IgG is a control. (C) Schematic diagrams of the full-length Nsp2 (aa1-1196) and truncated Nsp2 (Nsp2-N, aa1-405; Nsp2-M, aa323-844), all tagged with mCherry at the C-terminus. HV, Hypervariable region; TM, transmembrane domain; Nsp2-N, Nsp2 N-terminal; Nsp2-M, Nsp2 middle. (D) mCherry empty vector, and mCherry-tagged Nsp2, Nsp2-N and Nsp2-M were transfected into PAMs for 36 h, respectively. The transcription of TREM2 is shown, as measured by qRT-PCR. (E and F) The interaction of TREM2 with Nsp2, Nsp2-N, and Nsp2-M by Co-IP. 293T cells (E) or Marc-145 cells (F) were co-transfected with the indicated plasmids. The cell lysates were immunoprecipitated with a Myc antibody and immunoblots with Myc and mCherry antibodies are shown. GAPDH is shown as an internal control. Asterisks mark the expressed mCherry-fusion proteins of full-length or other truncated Nsp2. Data are representative of the results of three independent experiments.
(TIF)

S3 Fig TREM2 overexpression promotes CD163 expression. (A) Cell supernatant levels of sCD163 are shown, as determined by ELISA at 12, 24 and 36 hpi in PAMs with TREM2 knockdown. (B—D) PAMs were transfected with pcDNA3.1-control (vector) or pcDNA3.1-TREM2 for 24 h, and infected with PRRSV (MOI = 1) for an additional 24 h. The changes of protein levels of CD163, ADAM17, PRRSV N, and TREM2 are shown, as detected by western blot. GAPDH is shown as an internal control (B). Representative histograms from flow cytometry analysis of cell surface CD163 on PAMs when TREM2 is overexpressed (C). Positive cell ratio of cell surface CD163 based on analysis conditions in C (D). Data are representative of the results of three independent experiments (mean ± SE). Significant differences are indicated as follows: * (*P* < .05), ** (*P* < .01) and *** (*P* < .001).
(TIF)

S4 Fig TREM2 knockdown causes a reduction of CD163 mediated by cytokines to suppress virus replication via Syk/PI3K and TLR4/ NF-κB signaling. (A—H) PAMs with TREM2 knockdown were mock-treated or treated with R406 (5 μM), Wortmannin (1 μM) or BAY11-7082 (10 μM), respectively, then mock-infected or infected with PRRSV for 24 h. Gene expression of IL-1β (A), IL-8 (B), TNF-α (C), IFN-β (D), IFN-α (E), NF-κB (F), ADAM17 (G), and CD163 (H) are shown using qRT-PCR analysis. (I—K) PAMs were either infected with PRRSV or treated with TAK-242 (TLR4 inhibitor, 10 μM) or dexamethasone (100 nM) before infection in conditions of TREM2 knockdown. Transcriptional levels of CD163 are shown, as detected by qRT-PCR (I). The protein levels of CD163 and PRRSV N are shown, as detected by western blot (J and K). GAPDH is shown as an internal control. (L) Cells were treated with these inhibitors mentioned above in conditions of TREM2 knockdown. PRRSV N transcription was detected by qRT-PCR. Data are representative of the results of two independent experiments (mean ± SE). Significant differences are indicated as follows: * (*P* < .05), ** (*P* < .01) and *** (*P* < .001).
(TIF)

S5 Fig sTREM2 inhibits PRRSV replication. (A and B) PAMs were infected with PRRSV at different MOIs (0, 0.4, 0.8, 1.6 and 3.2) for 24 h (A) or infected with PRRSV (MOI = 1) for the indicated periods (0, 6, 12, 24 and 36 hpi) (B), sTREM2 production in cell supernatants was measured by ELISA. (C) Expression and purification of TREM2 in *Escherichia coli* BL21 cells. Lane 1 is the purified sTREM2 (18 kDa). Lane 2 is the control. M is the protein molecular weight marker. (D) Expression and purification of GFP protein in *Escherichia coli* BL21 cells. Lane 1 is the control. Lane 2 is the purified GFP protein (27 kDa). M is the protein molecular weight marker. (E) For the entry assay, Marc-145 cells were initially challenged with PRRSV (MOI = 5) for 3 h at 4°C. Then, unbound viral particles were removed, and cells were cultured at 37°C in the presence of various concentrations of sTREM2 or GFP protein (20 and 40 pg/mL) for 6 h. These proteins were added at 0, 2, or 4 h after the temperature shift to 37°C. After washing with PBS three times, cells were incubated for another 24 h at 37°C. Western blot was used to detect the expression of PRRSV N. Purified GFP protein serves as a negative control. (F and G) mCherry-tagged GP4 (F) or mCherry-tagged GP5 (G) was co-transfected with Myc-tagged full-length or other truncated TREM2 in HEK293T. IP assays with anti-Myc antibody were performed to determine their interaction, and the GP4-mCherry or GP5-mCherry was detected by IB with the anti-mCherry antibody. Asterisks mark the expressed Myc-fusion proteins of TREM2 fragments. Data are representative of the results of three independent experiments.
(TIF)

S1 Table Sequences of siRNAs used in this study. (DOCX)

S2 Table List of primers for qRT-PCR. (DOCX)

S1 Data All numerical values that were used to generate graphs and histograms. Excel spreadsheet contains the underlying numerical data and statistical analysis for Fig panels 1C, 1E, 1F, 1G, 1I, 1K, 1M, 1N, 2B, 2D, 4A, 4B, 4C, 4D, 4E, 4F, 4I, 5A, 5C, 5D, 6C, 6D, 6H, 8A, 8D, 8E, 8G, 8H, S1A, S1B, S2D, S3A, S3D, S4A, S4B, S4C, S4D, S4E, S4F, S4G, S4H, S4I, S4L, S5A, and S5B in separate sheets.
(XLSX)
