# Supplementary figures and images for: TREM2 suppresses the proinflammatory response to facilitate PRRSV infection via PI3K/NF-κB signaling
Source: PLoS Pathog. 2020 May 13;16(5):e1008543. doi: 10.1371/journal.ppat.1008543 (PMC7250469; doi:10.1371/journal.ppat.1008543)

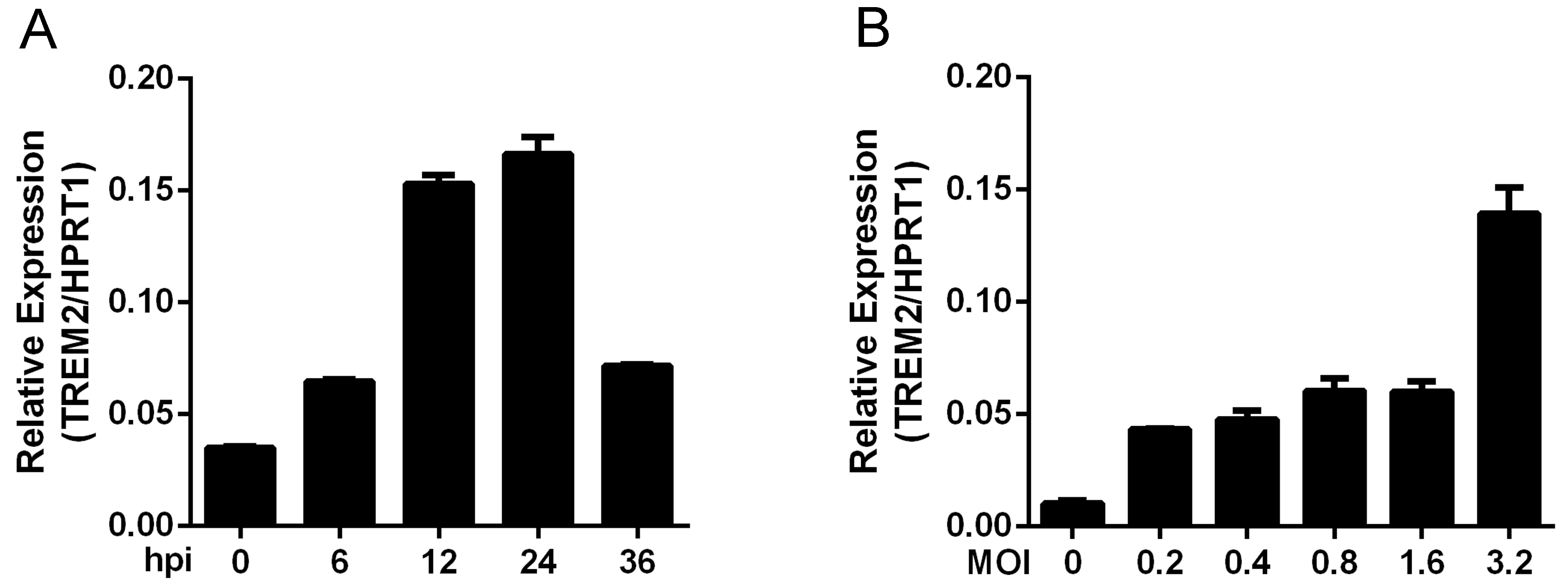

Supplement: S1 Fig — (A and B) PAMs were infected with PRRSV (MOI = 1) for the indicated time periods (0, 6, 12, 24 and 36 hpi) (A) or at different MOIs (0, 0.2, 0.4, 0.8, 1.6 and 3.2) for 24 h (B), transcriptional levels of TREM2 are shown, as detected by qRT-PCR. Data are representative of the results of three independent experiments (mean ± SE). (TIF) [file ppat.1008543.s001.tif]

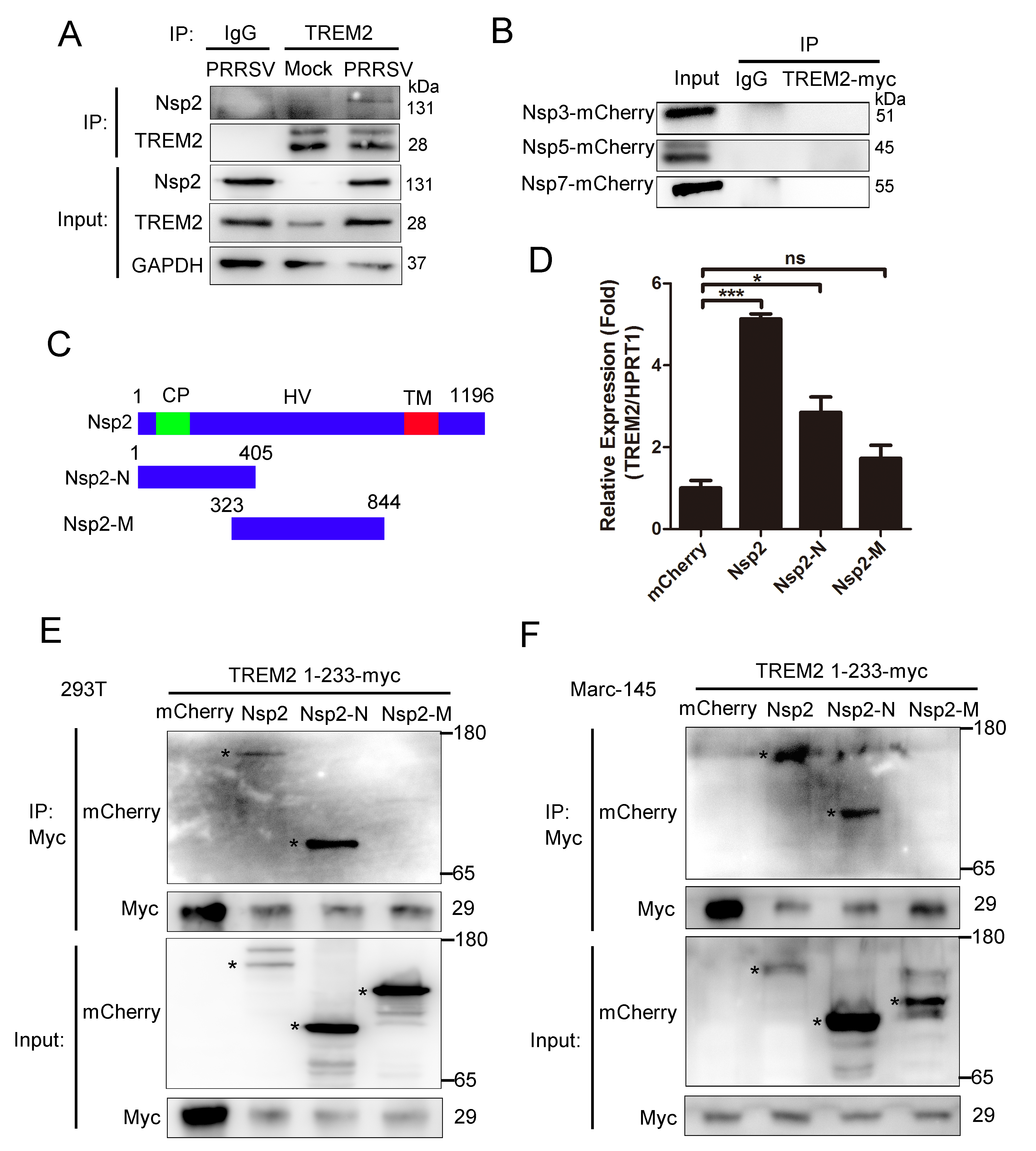

Supplement: S2 Fig — (A) PAMs were mock-infected or infected with PRRSV (MOI = 1) for 24 h. Cell lysates were immunoprecipitated with TREM2 antibody, and the immunoblots with Nsp2 and TREM2 antibodies are shown. GAPDH is shown as an internal control. (B) Myc-tagged TREM2 and mCherry-tagged Nsp3, Nsp5 or Nsp7 were co-transfected into HEK293T for 36 h. Cell lysates were immunoprecipitated with Myc or IgG antibodies and the immunoblots are shown with mCherry antibodies. IgG is a control. (C) Schematic diagrams of the full-length Nsp2 (aa1-1196) and truncated Nsp2 (Nsp2-N, aa1-405; Nsp2-M, aa323-844), all tagged with mCherry at the C-terminus. HV, Hypervariable region; TM, transmembrane domain; Nsp2-N, Nsp2 N-terminal; Nsp2-M, Nsp2 middle. (D) mCherry empty vector, and mCherry-tagged Nsp2, Nsp2-N and Nsp2-M were transfected into PAMs for 36 h, respectively. The transcription of TREM2 is shown, as measured by qRT-PCR. (E and F) The interaction of TREM2 with Nsp2, Nsp2-N, and Nsp2-M by Co-IP. 293T cells (E) or Marc-145 cells (F) were co-transfected with the indicated plasmids. The cell lysates were immunoprecipitated with a Myc antibody and immunoblots with Myc and mCherry antibodies are shown. GAPDH is shown as an internal control. Asterisks mark the expressed mCherry-fusion proteins of full-length or other truncated Nsp2. Data are representative of the results of three independent experiments. (TIF) [file ppat.1008543.s002.tif]

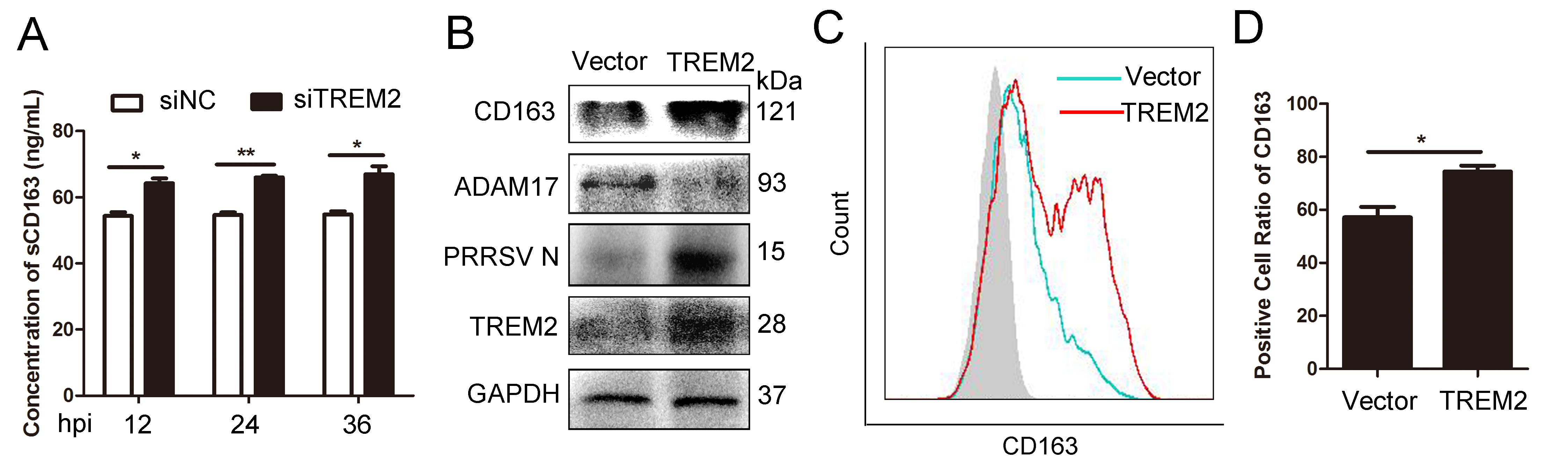

Supplement: S3 Fig — (A) Cell supernatant levels of sCD163 are shown, as determined by ELISA at 12, 24 and 36 hpi in PAMs with TREM2 knockdown. (B—D) PAMs were transfected with pcDNA3.1-control (vector) or pcDNA3.1-TREM2 for 24 h, and infected with PRRSV (MOI = 1) for an additional 24 h. The changes of protein levels of CD163, ADAM17, PRRSV N, and TREM2 are shown, as detected by western blot. GAPDH is shown as an internal control (B). Representative histograms from flow cytometry analysis of cell surface CD163 on PAMs when TREM2 is overexpressed (C). Positive cell ratio of cell surface CD163 based on analysis conditions in C (D). Data are representative of the results of three independent experiments (mean ± SE). Significant differences are indicated as follows: * (P < .05), ** (P < .01) and *** (P < .001). (TIF) [file ppat.1008543.s003.tif]

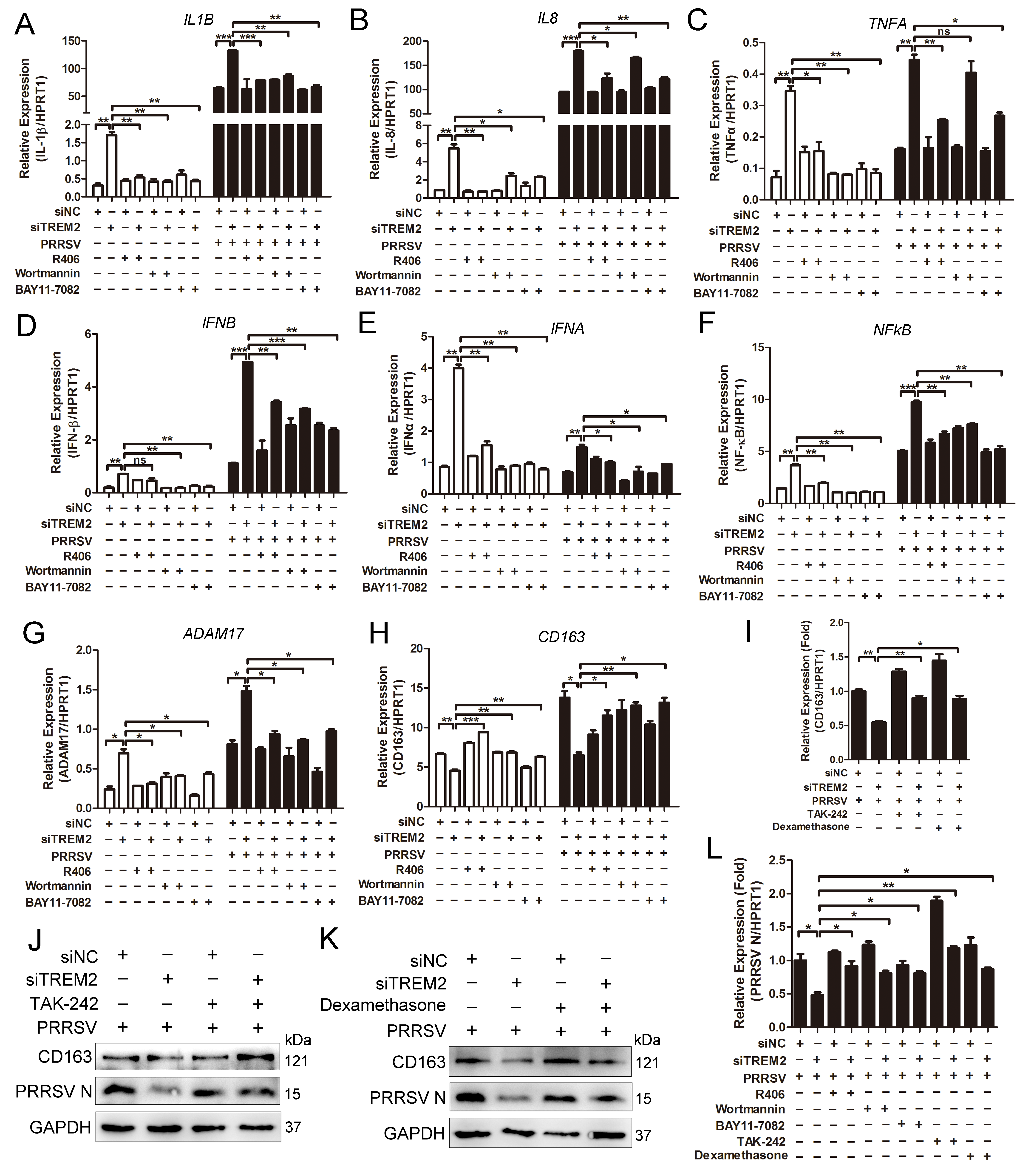

Supplement: S4 Fig — (A—H) PAMs with TREM2 knockdown were mock-treated or treated with R406 (5 μM), Wortmannin (1 μM) or BAY11-7082 (10 μM), respectively, then mock-infected or infected with PRRSV for 24 h. Gene expression of IL-1β (A), IL-8 (B), TNF-α (C), IFN-β (D), IFN-α (E), NF-κB (F), ADAM17 (G), and CD163 (H) are shown using qRT-PCR analysis. (I—K) PAMs were either infected with PRRSV or treated with TAK-242 (TLR4 inhibitor, 10 μM) or dexamethasone (100 nM) before infection in conditions of TREM2 knockdown. Transcriptional levels of CD163 are shown, as detected by qRT-PCR (I). The protein levels of CD163 and PRRSV N are shown, as detected by western blot (J and K). GAPDH is shown as an internal control. (L) Cells were treated with these inhibitors mentioned above in conditions of TREM2 knockdown. PRRSV N transcription was detected by qRT-PCR. Data are representative of the results of two independent experiments (mean ± SE). Significant differences are indicated as follows: * (P < .05), ** (P < .01) and *** (P < .001). (TIF) [file ppat.1008543.s004.tif]

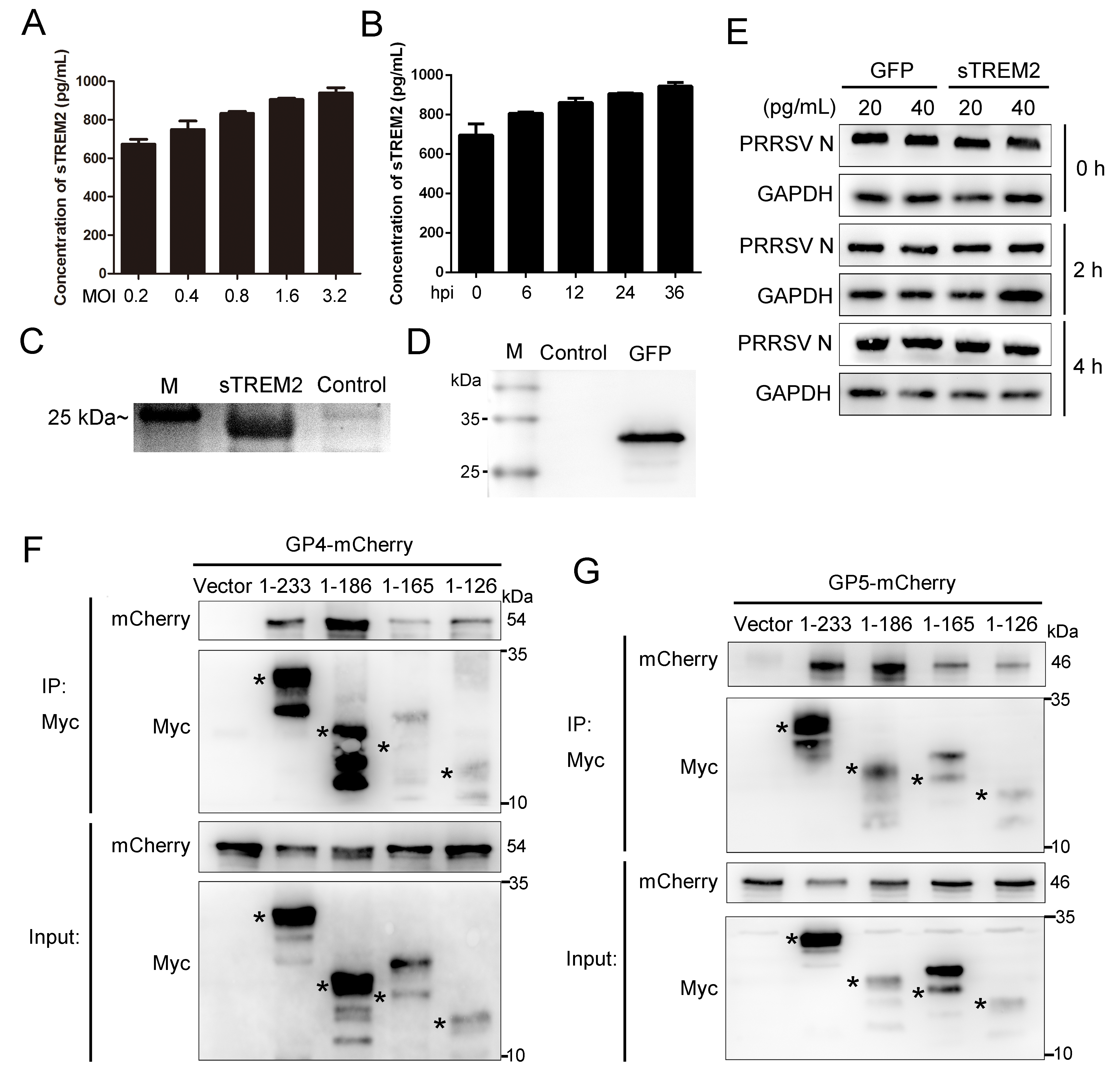

Supplement: S5 Fig — (A and B) PAMs were infected with PRRSV at different MOIs (0, 0.4, 0.8, 1.6 and 3.2) for 24 h (A) or infected with PRRSV (MOI = 1) for the indicated periods (0, 6, 12, 24 and 36 hpi) (B), sTREM2 production in cell supernatants was measured by ELISA. (C) Expression and purification of TREM2 in Escherichia coli BL21 cells. Lane 1 is the purified sTREM2 (18 kDa). Lane 2 is the control. M is the protein molecular weight marker. (D) Expression and purification of GFP protein in Escherichia coli BL21 cells. Lane 1 is the control. Lane 2 is the purified GFP protein (27 kDa). M is the protein molecular weight marker. (E) For the entry assay, Marc-145 cells were initially challenged with PRRSV (MOI = 5) for 3 h at 4°C. Then, unbound viral particles were removed, and cells were cultured at 37°C in the presence of various concentrations of sTREM2 or GFP protein (20 and 40 pg/mL) for 6 h. These proteins were added at 0, 2, or 4 h after the temperature shift to 37°C. After washing with PBS three times, cells were incubated for another 24 h at 37°C. Western blot was used to detect the expression of PRRSV N. Purified GFP protein serves as a negative control. (F and G) mCherry-tagged GP4 (F) or mCherry-tagged GP5 (G) was co-transfected with Myc-tagged full-length or other truncated TREM2 in HEK293T. IP assays with anti-Myc antibody were performed to determine their interaction, and the GP4-mCherry or GP5-mCherry was detected by IB with the anti-mCherry antibody. Asterisks mark the expressed Myc-fusion proteins of TREM2 fragments. Data are representative of the results of three independent experiments. (TIF) [file ppat.1008543.s005.tif]
