# Supplementary material for: TREM2 suppresses the proinflammatory response to facilitate PRRSV infection via PI3K/NF-κB signaling
Source: PLoS Pathog. 2020 May 13;16(5):e1008543. doi: 10.1371/journal.ppat.1008543 (PMC7250469; doi:10.1371/journal.ppat.1008543)
Supplement: S1 Table — (DOCX) [file ppat.1008543.s006.docx]

**S1 Table. Sequences of siRNAs used in this study.**

| siRNA | siRNA sequence (sense 5′-3′) | siRNA sequence (anti-sense 5′-3′) |
| --- | --- | --- |
| siTREM2-1 | UUGAAGAUUGCGCAGCGUAAUGGUG | CACCAUUACGCUGCGCAAUCUUCAA |
| siTREM2-2 | CCAAGAGCUUCCAGGAUGUUCAGAU | AUCUGAACAUCCUGGAAGCUCUUGG |
| siTREM2-3 | GCGUCUUUCUCAGCAAGCUUCUAGU | ACUAGAAGCUUGCUGAGAAAGACGC |
| siNC | UUCUCCGAACGUGUCACGUTTAUUA | ACGUGACACGUUCGGAGAATTUAGG |
