# Supplementary material for: TREM2 suppresses the proinflammatory response to facilitate PRRSV infection via PI3K/NF-κB signaling
Source: PLoS Pathog. 2020 May 13;16(5):e1008543. doi: 10.1371/journal.ppat.1008543 (PMC7250469; doi:10.1371/journal.ppat.1008543)
Supplement: S2 Table — (DOCX) [file ppat.1008543.s007.docx]

**S2 Table. List of primers for qRT-PCR.**

| Primer^a^ | Sequence (5'-3')^b^ |
| --- | --- |
| ORF7 (N)-F | AAAACCAGTCCAGAGGCAAG |
| ORF7 (N)-R | CGGATCAGACGCACAGTATG |
| TREM2-F | CCCACCTGGCTGTTGTCCTT |
| TREM2-R | TCGCTACCGTGGAGGCTCTG |
| ADAM17-F | GCACAGGTAATAGCAGTGAGTGC |
| ADAM17-R | CACACAATGGACAAGAATGCTG |
| CD163-F | ATTCATCATCCTCGGACCCAT |
| CD163-R | CCCAGCACAACGACCACCT |
| IL-1β-F | CCCAAAAGTTACCCGAAGAGG |
| IL-1β-R | TCTGCTTGAGAGGTGCTGATG |
| IL-6-F | CTGCTTCTGGTGATGGCTACTG |
| IL-6-R | GGCATCACCTTTGGCATCTT |
| IL-8-F | AGTTTTCCTGCTTTCTGCAGCT |
| IL-8-R | TGGCATCGAAGTTCTGCACT |
| IL-10-F | TGAGAACAGCTGCATCCACTTC |
| IL-10-R | TCTGGTCCTTCGTTTGAAAGAAA |
| TNF-α-F | ACTCGGAACCTCATGGACAG |
| TNF-α-R | AGGGGTGAGTCAGTGTGACC |
| IFN-α-F | TCCAGCTCTTCAGCACAGAG |
| IFN-α-R | AGCTGCTGATCCAGTCCAGT |
| IFN-β-F | AGCACTGGCTGGAATGAAACCG |
| IFN-β-R | CTCCAGGTCATCCATCTGCCCA |
| TLR4-F | CCTGCCTGTGCTGAGTTTCA |
| TLR4-R | AAGGTGAGAACTGACGCACTAATG |
| MyD88-F | GGCAGCTGGAACAGACCAA |
| MyD88-R | GGTGCCAGGCAGGACATC |
| NF-κB-F | TCGCTGCCAAAGAAGGACAT |
| NF-κB-R | AGCGTTCAGACCTTCACCGT |
| ERK-F | GACGCAGCACCTCAGCAA |
| ERK-R | CACGGGCCAACCCAAAGT |
| HPRT1-F | TGGAAAGAATGTCTTGATTGTTGAAG |
| HPRT1-R | ATCTTTGGATTATGCTGCTTGACC |

^a^F: forward primer, R: reverse primer.

^b^Pig gene sequences and PRRSV gene sequences were downloaded from GenBank.
